# Regulatory Role of Adrenomedullin in Hypoxic Adaptation of Yak Subcutaneous Preadipocytes

**DOI:** 10.3390/ani16162531

**Published:** 2026-08-13

**Authors:** Su Shan, Hui Jiang, Jincheng Zhong, Yuqing Zhang, Heru Zhang, Zhixin Chai

**Affiliations:** 1 Sichuan Qinghai-Tibet Plateau Herbivore Livestock Engineering Technology Center, Southwest Minzu University, Chengdu 610225, China; shansu2022@163.com (S.S.); zhongjincheng518@126.com (J.Z.); 15648007949@163.com (Y.Z.); 15642243411@139.com (H.Z.); 2 State Key Laboratory of Hulless Barley and Yak Germplasm Resources and Genetic Improvement, Institute of Animal Science and Veterinary Research, Tibet Academy of Agricultural and Animal Husbandry Sciences, Lhasa 850000, China; jh03201119@163.com

**Keywords:** adrenomedullin, yak, subcutaneous preadipocytes, hypoxia adaptation

## Abstract

High-altitude hypoxic environments constrain yaks’ survival capacity and metabolic adaptability. Subcutaneous adipose tissue helps yaks withstand cold and resist hypoxic stress, and ADM participates in cellular stress and metabolic regulation. To explore the regulatory role of ADM in the hypoxia adaptation of yak subcutaneous preadipocytes in high-altitude environments, we established three cell culture groups (normoxia, physiological hypoxia, and hypoxia). We further systematically examined cell proliferation, apoptosis, adenosine triphosphate (ATP) production, and lipid metabolic markers, and we performed transcriptome sequencing to reveal their regulatory effects. ADM exhibited a bidirectional regulatory effect: Low doses alleviated hypoxia-induced cell damage and restored energy and lipid synthesis, whereas high doses, in conjunction with hypoxia, activated multiple metabolic pathways and altered cellular energy-supply patterns. Under physiological hypoxia, cells emphasized metabolic regulation, while under hypoxia, they prioritized stress defense. This study demonstrates that under hypoxic stress, ADM regulates the survival and physiological function of yak subcutaneous preadipocytes in a dose-dependent manner, with cells relying on staged stress-defense and metabolic-remodeling responses to achieve adaptation to hypoxia, providing important experimental and theoretical insights into the mechanisms of high-altitude hypoxia adaptation in yaks.

## Simple Summary

In high-altitude areas, low oxygen availability disrupts the growth, survival, and energy supply of yak subcutaneous preadipocytes and reduces their ability to adapt to the plateau environment. Subcutaneous adipose tissue helps yaks resist severe cold, and adrenomedullin (ADM) regulates cellular responses to hypoxic stimuli while maintaining metabolic stability. In this study, three oxygen environments were established: normoxia (21% O_2_), physiological hypoxia (10% O_2_), and hypoxia (1% O_2_), each combined with different ADM doses. Cell growth status was observed, and energy-metabolism-related indicators were analyzed. The results show that hypoxia hinders the growth of yak subcutaneous preadipocytes, accelerates cellular damage, and reduces energy and lipid accumulation. ADM exhibits a dose-dependent, bidirectional regulatory effect on yak subcutaneous preadipocytes under hypoxic stress. Additionally, yak subcutaneous preadipocytes adjust their defense mechanisms and energy supply in segments according to oxygen concentrations to adapt to hypoxic conditions. This study reveals the mechanism of yak adaptation to high-altitude hypoxia and provides practical references for plateau livestock farming and breeding resilient livestock.

## 1. Introduction

The yak (*Bos grunniens*) is a unique bovine species found primarily on the Qinghai–Tibet Plateau and adjacent regions at altitudes above 3000 m. It can adapt to extreme ecological conditions marked by severe cold, hypoxia, long cold seasons, short warm seasons, and limited forage. As a result, yaks hold irreplaceable ecological and economic value within their distribution areas. Long-term habitation in high-altitude regions, together with geographic isolation and natural domestication, has shaped stable physiological compensatory mechanisms and metabolic adaptation strategies in yaks. Over time, they have also developed unique adaptive traits in cardiopulmonary structures and oxygen-transport capacity. Therefore, yaks are a model species for studying mammalian adaptation to high-altitude hypoxia [1]. Adipose tissue is a vital energy-storage and endocrine organ that plays a key role in maintaining body heat, energy storage, and lipid metabolism [2], all of which are pivotal for survival in cold, high-altitude environments. Compared with visceral adipose tissue, subcutaneous adipose tissue is more widely distributed and exhibits greater metabolic plasticity. It senses external signals, such as ambient temperature, partial pressure of oxygen, and nutritional status, and adjusts metabolic regulation and nutrient use accordingly [3]. As the basic functional units of subcutaneous adipose tissue, subcutaneous preadipocytes have proliferation activity, mitochondrial function, and metabolic levels that directly determine the body’s metabolic function. Together, these traits influence the yak’s adaptability to high-altitude, hypoxic environments. It should be noted that mature adipocytes are terminally differentiated cells with negligible proliferative potential, while subcutaneous preadipocytes retain robust proliferative ability. Therefore, studying subcutaneous preadipocyte proliferation and metabolism in yaks under hypoxic conditions is of great value, as it can help clarify how plateau livestock adapt to their challenging environments.

Oxygen is indispensable for normal cellular activities and serves as the primary substrate for mitochondrial aerobic respiration, the citric acid cycle, and oxidative phosphorylation. Thus, cellular energy metabolism depends greatly on a stable environmental oxygen concentration [4]. Under normoxic (21% O_2_) conditions in mammals, adipocytes primarily rely on oxidative phosphorylation to efficiently generate ATP, ensuring the normal progression of various cellular processes. However, under hypoxic (1% O_2_) conditions, mitochondrial respiratory function is impaired, leading to insufficient energy production. To adapt, cells undergo metabolic reprogramming by enhancing glycolysis, which reduces ATP production and leads to lactate accumulation; this disrupts cellular energy homeostasis [5]. Furthermore, studies have shown that hypoxic conditions can induce upregulation of uncoupling protein 1 (UCP1) and uncoupling protein 3 (UCP3) in skeletal muscles and subcutaneous adipose tissues. These changes are accompanied by metabolic remodeling, indicating that subcutaneous adipose tissue plays an important role in the adaptation of energy metabolism and cold tolerance in animals under hypoxic conditions [6]. In this adaptation, hypoxia-inducible factor (HIF) acts as the primary transcription factor mediating the effects of oxygen receptors in animal tissues and organs. The HIF signaling pathway widely regulates lipid anabolism, fatty acid turnover, and lipid droplet biogenesis, but sustained abnormal activation of HIF is a major contributor to metabolic dysfunction [7]. Therefore, elucidating how transcriptional regulatory networks under hypoxic stress affect the physiology and glucolipid metabolism of subcutaneous adipocytes may clarify the intrinsic mechanisms of hypoxia-mediated metabolic reprogramming in adipocytes.

Given the complex oxygen microenvironment of adipose tissue and following the standard nomenclature in hypoxia research, we established three fractional inspired oxygen (FiO_2_) gradients in this study to simulate distinct in vivo oxygen scenarios of yak subcutaneous adipose tissue. In this study, 21% FiO_2_ was set as the normoxic control, corresponding to sea-level atmospheric oxygen with a gas-phase oxygen partial pressure (PO_2_) of ≈21.28 kPa. Then, 10% FiO_2_ was designed as the physiological hypoxia group, matching the ambient oxygen level at 3500 m—the main long-term habitat of yaks on the Qinghai–Tibet Plateau—with a gas-phase PO_2_ of ≈10.13 kPa, which represents the routine systemic oxygen background of yak subcutaneous adipose tissue in vivo. Our study also used 1% FiO_2_ as the severe hypoxia group, which does not correspond to any natural atmospheric altitude but recapitulates the intrinsic severe interstitial hypoxic microenvironment inside adipose tissue. Subcutaneous adipose tissue is characterized by sparse microvascular networks and high oxygen consumption by adipocytes, forming a steep oxygen gradient from perivascular regions to distant interstitial zones; in vivo oxygen electrode measurements have further confirmed that interstitial PO_2_ in adipocyte-rich, vascular-poor fat depots can drop to 1–2 kPa [8,9], which is highly consistent with the gas-phase PO_2_ of our 1% FiO_2_ condition (≈1.01 kPa). Notably, the two hypoxic gradients represent completely distinct physiological scenarios: 10% FiO_2_ mimics the macroscopic ambient atmospheric hypoxia at the natural living altitude of yaks, while 1% FiO_2_ recapitulates severe local interstitial hypoxia within adipose tissue independent of external altitude. This gradient design allows us to systematically explore the differential regulatory effects of different hypoxic patterns on the biological function of yak subcutaneous preadipocytes.

ADM is a highly conserved, multifunctional natural peptide. It was first found in adrenal medullary tumor tissue [10]. Studies show that ADM is not only found in adrenal tissue but is also stable in fat tissue [11]. ADM binds to its specific receptors in the same cell (autocrine) or in nearby cells (paracrine), helping regulate processes such as cell growth, cell death, mitochondrial structure and function, the balance of oxidative stress, and sugar and fat metabolism [12]. In particular, the regulatory effects of ADM on adipocyte energy metabolism and its hypoxia-responsive properties have attracted widespread research interest in recent years [13]. ADM is a typical hypoxia-inducible bioactive peptide, for which its expression is significantly upregulated via the HIF-1α-dependent signaling pathway under low-oxygen microenvironments [14]. Cumulative evidence further confirms that ADM serves as a pivotal regulatory factor governing adipocyte proliferation, adipogenic differentiation, and metabolic homeostasis [15]. Specifically, ADM can remodel the balance between glycolysis and oxidative phosphorylation in adipocytes through receptor-mediated downstream signaling cascades to counteract hypoxic stress, consequently altering mitochondrial respiratory capacity and cellular energy production [16]. Although the hypoxic metabolic regulatory function of ADM has been partially characterized in several model organisms, its exact physiological roles in yaks remain largely unclear. Understanding how ADM regulates yak energy use under hypoxic conditions may reveal key steps animals take to maintain stable energy at high altitudes. Therefore, it is critical to screen candidate genes potentially involved in ADM-mediated hypoxic responses in yak subcutaneous preadipocytes.

## 2. Materials and Methods

### 2.1. Animal Materials

All experimental yaks were obtained from Hongyuan County, Aba Tibetan, and Qiang Autonomous Prefecture, Sichuan Province, a typical high-altitude pastoral region on the Qinghai–Tibet Plateau. The sampling site has an average altitude of approximately 3600 m. These yaks were raised under a traditional alpine grazing feeding mode, freely feeding on natural alpine meadow herbage. All experimental individuals were screened to be 36–48 months old with consistent body weight and good health status to minimize the interference of individual differences and habitat environmental factors on adipocyte metabolic phenotypes. After anesthesia, the yaks were euthanized via exsanguination in full compliance with animal welfare regulations. Subcutaneous abdominal fat tissue was promptly harvested, and the tissue surface was cleaned. The samples were then placed in pre-chilled phosphate-buffered saline (PBS) containing 2% penicillin–streptomycin (P/S) solution and transported at low temperatures to the cell culture laboratory. All animal procedures in this experiment strictly adhered to the Guidelines for the Ethical Review of Laboratory Animals and the Guiding Opinions on the Humane Treatment of Laboratory Animals. The procedures were reviewed and approved by the Animal Care and Use Committee of Southwest Minzu University and conducted in accordance with relevant guidelines and regulations established by the local animal ethics committee.

### 2.2. Isolation and Culture of Yak Subcutaneous Preadipocytes

Subcutaneous adipose tissue was isolated from the yak abdomen and rinsed 3 to 4 times with PBS containing 3% P/S. Hair, blood vessels, and fascia were carefully removed, and the cleaned tissue was minced into small pieces with a volume of 1–3 mm^3^. The tissue fragments were incubated with a mixture of collagenase Type I and Type II in a shaking incubator at 37 °C for 3–4 h. Following enzymatic digestion, the homogenate was sequentially filtered through 70 μm and 40 μm cell strainers to collect the filtrate, and the digestion reaction was terminated immediately. The filtrate was centrifuged at 1800 r/min for 10 min, and the supernatant was discarded to retain the cell pellets. The pellets were resuspended in a red blood cell lysis buffer and incubated at room temperature for 10 min; then, they were centrifuged at 2000 r/min for 10 min. After removing the supernatant, the cell precipitates were resuspended in fresh PBS and centrifuged again at 1800 r/min for 5 min. The harvested cells were seeded into culture dishes and cultured in an incubator (Thermo Fisher Scientific, Waltham, MA, USA) at 37 °C with 5% CO_2_. The culture medium was refreshed the next day and thereafter replaced every 2 days until cell passaging or cryopreservation. The complete culture medium consisted of 89% DMEM, 10% fetal bovine serum (FBS), and 1% pen-strep. The differentiation medium was prepared by supplementing the complete medium with oleic acid (Merck, Rahway, NJ, USA) to a final concentration of 100 μM. PBS, DMEM, red blood cell lysis buffer, collagenase Type I, collagenase Type II, and penicillin–streptomycin were all purchased from Gibco (Thermo Fisher Scientific, Waltham, NY, USA).

### 2.3. Treatment of Yak Subcutaneous Preadipocytes

After cell seeding and attachment, the medium was replaced with a complete medium containing adrenomedullin. The ADM stock solution was prepared by dissolving ADM in dimethyl sulfoxide (DMSO) to a concentration of 1 mM, aliquoted, and stored at −80 °C in the dark. For the experiment, first, the 1 mM DMSO primary stock solution was diluted with complete culture medium to prepare a 1 μM intermediate working solution. Then, the above 1 μM medium-based intermediate working solution was added into the complete culture medium and diluted to final treatment concentrations of 100 nM, 10 nM, 1 nM, and 100 pM, respectively, to investigate ADM effects on the proliferation, differentiation, apoptosis, and energy metabolism of yak subcutaneous preadipocytes. A blank control group was also established, with three biological replicates in each group. All treatment groups and the control group were cultured in incubators with different oxygen concentrations. This experiment set three culture gradients based on the FiO_2_, namely 21% O_2_, 10% O_2_, and 1% O_2_. The corresponding PO_2_ under standard atmospheric pressure (101.325 kPa) was calculated for each group: 21% O_2_ (PO_2_ ≈ 21.28 kPa) served as the normoxic control, representing the standard sea-level atmospheric oxygen environment for routine in vitro cell cultures. Then, 10% O_2_ (PO_2_ ≈ 10.13 kPa) simulated the ambient atmospheric oxygen conditions at 3500 m, the primary long-term habitat of plateau yaks, which matches the moderate oxygen tension that yak adipose tissue chronically adapts to in vivo. In this study, 1% O_2_ (PO_2_ ≈ 1.01 kPa) was not used to simulate ultra-high atmospheric altitude conditions. Subcutaneous adipose tissue features sparse microvascular networks and limited oxygen perfusion; oxygen is continuously consumed during diffusion from capillaries, forming a steep intratissue oxygen gradient. Therefore, 1% O_2_ was adopted to recapitulate the intrinsic severe interstitial hypoxic microenvironment within yak subcutaneous adipose tissue rather than extreme plateau atmospheric hypoxia.

### 2.4. Identification of Yak Subcutaneous Preadipocytes

Subcutaneous preadipocytes were seeded into culture dishes and cultured at 37 °C in a humidified atmosphere containing 5% CO_2_ until reaching 80% confluence before subsequent experiments. After cell adherence and stable growth, cell morphology was observed and photographed under an inverted microscope. To induce adipogenic differentiation, yak subcutaneous preadipocytes were seeded into 6-well plates at a density of 5 × 10^4^ cells/cm^2^. After the cells adhered, the complete medium was discarded and replaced with medium containing 100 μM oleic acid, followed by routine culture for 6 days. Total RNA was collected from both preadipocytes and differentiated cells, and marker genes specific to each were selected for quantitative polymerase chain reaction (qPCR). *CD34* and *ITGB1* were used as preadipocyte markers, while *PPARγ* and *FABP4* served as differentiation markers.

### 2.5. Cell Counting Kit-8 (CCK-8)

Cells were seeded at 6 × 10^3^ cells/cm^2^ in a 96-well plate with 100 μL of complete medium. After the cells adhered under standard conditions, the medium was replaced with complete medium containing ADM at varying concentrations (100 pM, 1 nM, 10 nM, and 100 nM). A blank control group and a zero-calibration well were included, each with six replicates. Plates were incubated in 1%, 10%, or 21% O_2_. At 0, 24, 48, 72, and 96 h, the culture medium was replaced with 100 μL of medium containing 10% Cell Counting Kit-8 (SparkJade, Jinan, China), and samples were incubated in the dark for 1–2 h. OD was measured at 450 nm using an enzyme-linked immunosorbent assay (ELISA) detector (Thermo Fisher Scientific, USA).

### 2.6. Flow Cytometry

Seed 5 × 10^4^ cells/cm^2^ into a 6-well culture plate. Add 2 mL of complete medium to each well and incubate the plate in a standard incubator for 24 h. Once the cells are fully adherent, replace the medium with a complete medium containing different concentrations of ADM. Use three biological replicates per group. Place the cells in incubators set to three oxygen levels (1% O_2_, 10% O_2_, and 21% O_2_) and incubate at a constant temperature for 48 h. Collect cell samples from each group. Perform double staining using the Annexin-V/PI apoptosis detection kit (UElandy, Suzhou, China). Collect data with a flow cytometer, analyze with FlowJo software (v10.8.1), and calculate the apoptosis rate from the cell count in the apoptosis region.

### 2.7. Cell Scratch Test

Seed cells at a density of 5 × 10^4^ cells/cm^2^ in a 6-well culture plate. After 24 h, use a 200 μL sterile pipette tip to make a vertical scratch in the center of each well, creating a uniform area of injury. Discard the original serum-containing medium. Gently wash the cells three times with PBS to remove cell debris. Add serum-free medium with different concentrations of ADM (0 pM, 100 pM, 1 nM, and 10 nM). Test each concentration in three replicates. Incubate plates at the required oxygen concentrations. Cell migration and healing were observed at 0, 24, 48, and 72 h, and the results were photographed and saved.

### 2.8. BODIPY and Oil Red O Staining

After adding different concentrations of ADM to the complete medium, place the plates in incubators with different oxygen concentrations. Discard the medium and wash the cells three times with PBS. Add 4% paraformaldehyde (Biyuntian, Hefei, China) and fix the cells at room temperature for 30 min. Add a working solution of Oil Red O or BODIPY (Biyuntian, Hefei, China) to each well in the specified proportion. Incubate at room temperature in the dark for 20 min. Gently rinse the cells four times with PBS. Photograph the results using an inverted fluorescence microscope (Carl Zeiss, Oberkochen, Germany). After the cells are fully stained, add 1 mL of 60% isopropanol solution to each well and mix by pipetting at room temperature. Aspirate 200 μL of the solution from each well into a 96-well plate and measure absorbance at 510 nm using an enzyme-linked immunoassay detector. Set up three replicates for each experimental group.

### 2.9. Determination of Glucose, Lactate, and ATP Contents

After cells adhered in 6-well plates, discard the complete medium and replace it with varying concentrations of ADM. Incubate the cells for 48 h at different oxygen concentrations in cell culture incubators. After incubation, harvest and sonicate the cells; then, use an ATP assay kit (Bainianshengwu, Chongqing, China) to measure ATP levels in each group. Collect 200 μL of culture medium from each well, and measure glucose contents using the Glucose Assay Kit (Bainianshengwu, Chongqing, China) and lactate contents using the Lactate Assay Kit (Bainianshengwu, Chongqing, China). Each experimental group was set up with three replicates.

### 2.10. Determination of Triglyceride (TG) and Free Fatty Acid (FFA) Contents

After the cells were seeded and attached, they were differentiated with oleic acid for 6 d. The culture medium was then replaced with a medium containing different concentrations of ADM. The cells were incubated at different oxygen concentrations for 48 h and then harvested and sonicated. Intracellular TG levels were measured using a triglyceride assay kit (Bainianshengwu, Chongqing, China). Separately, 200 μL of culture medium was collected from each well to measure extracellular FFA levels with a free fatty acid assay kit (Bainianshengwu, Chongqing, China). Each experimental group included three replicates.

### 2.11. Transcriptomic Sequencing, Bioinformatics Analysis, and Gene Expression Validation

A total of 12 treatment groups were set for RNA sequencing using a 3 × 4 full factorial design, covering three oxygen gradients (normoxia, physiological hypoxia, and hypoxia) and four ADM concentrations (0 pM vehicle control, 100 pM, 1 nM, and 10 nM), with all groups completing library construction and sequencing successfully. N represents the normoxic group, P represents the physiological hypoxia (physioxia) group, and H represents the hypoxic group. Total RNA was extracted from subcutaneous preadipocytes treated with different oxygen and ADM concentrations using the TRIzol method. mRNA was enriched via Oligo(dT) magnetic beads, fragmented, and reverse-transcribed to double-stranded cDNA. Following end repair, A-tailing, adapter ligation, and PCR amplification, qualified cDNA libraries were sequenced on the DNBSEQ-T7 platform based on DNA nanoball (DNB) sequencing technology. Raw sequencing reads were subjected to quality control with FastQC and trimmed by Trimmomatic to generate clean reads. Clean reads were aligned to the yak reference genome (GCF_027580195.1) using HISAT2. Gene expression abundance was quantified as FPKM by StringTie. Differentially expressed genes (DEGs) were screened with DESeq2 (|log_2_FC| ≥ 1, FDR-adjusted *p* < 0.05), followed by GO and KEGG enrichment analyses. Protein–protein interaction networks of DEGs were built using the STRING database, and core hub genes were visualized with Cytoscape (v3.9.1). At the same time, total RNA was extracted from cells 48 h post-treatment using the SPARKeasy Cell RNA Rapid Extraction Kit (SparkJade, Jinan, China). RNA concentration and purity were measured using a spectrophotometer. Genomic DNA was removed; then, RNA was reverse-transcribed with the PrimeScript™ RT Kit (Takara, Otsu, Shiga, Japan). qPCR amplification was performed using TB Green^®^ Premix Ex Taq™ II (Takara, Otsu, Shiga, Japan). UXT served as the internal control gene. The relative mRNA expression levels of the target genes were calculated using the 2^−ΔΔCT^ method. All primers were designed and synthesized using Primer5 software; specific sequences are shown in Table S1. Data analysis and visualization were conducted using GraphPad Prism 8.0. Comparisons between two groups used an independent samples *t*-test, while comparisons among multiple groups used one-way analysis of variance (ANOVA). Multiple comparisons were analyzed with Duncan’s multiple range test. Data are presented as mean ± SEM. Statistical significance is indicated as follows: * *p* < 0.05, ** *p* < 0.01, and *** *p* < 0.001; ns indicates no significant difference.

### 2.12. Experimental Flowchart

**Figure 1:**
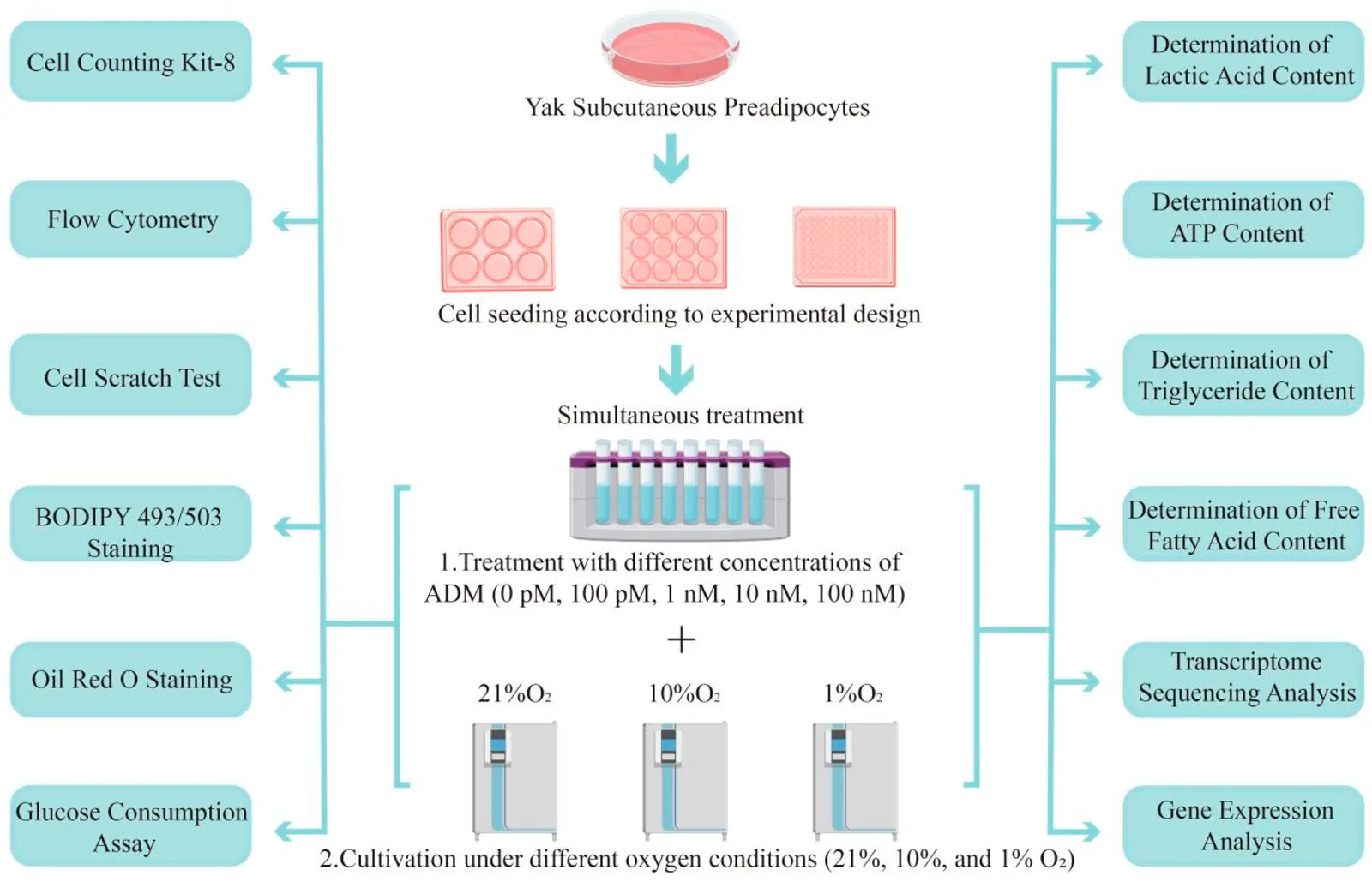
Experimental flowchart.

The overall experimental design and research workflow of this study are summarized in Figure 1, including cell treatment conditions, detection indicators and omics analysis strategies.

## 3. Results

### 3.1. Culture and Adipogenic Differentiation of Yak Subcutaneous Preadipocytes

**Figure 2:**
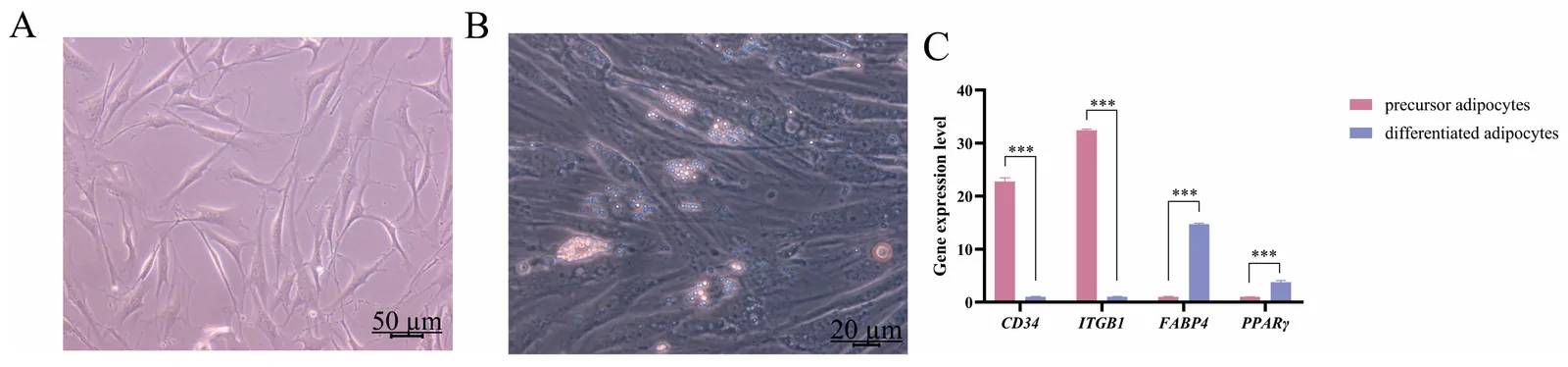
Culture and characterization of yak subcutaneous preadipocytes. (**A**) Yak subcutaneous preadipocytes under a light microscope (scale bar = 50 μm). (**B**) The appearance of differentiated yak subcutaneous preadipocytes under a light microscope (scale bar = 20 μm). (**C**) mRNA levels of marker genes in precursor and differentiated cells; data are the mean ± SEM. Asterisks show statistical significance: *** *p* < 0.001; ns means not significant. Each group had three replicates (*n* = 3).

Inverted microscopy showed that subcutaneous preadipocytes were morphologically uniform, spindle-shaped with tapered ends, and firmly attached to the substrate (Figure 2A). After induction of differentiation, the cells exhibited refractive lipid droplets (Figure 2B). Differential expression analysis revealed that proliferation markers *CD34* and *ITGB1* were significantly higher in precursor adipocytes (*p* < 0.001), while adipogenic differentiation markers *FABP4* and *PPARγ* were significantly upregulated in differentiated cells (Figure 2C). These gene expression patterns align with known changes during adipocyte differentiation, supporting the reliability of this subcutaneous preadipocyte model.

### 3.2. Effects of Hypoxic Stress on Proliferation, Apoptosis, and Energy Metabolism of Yak Subcutaneous Preadipocytes

**Figure 3:**
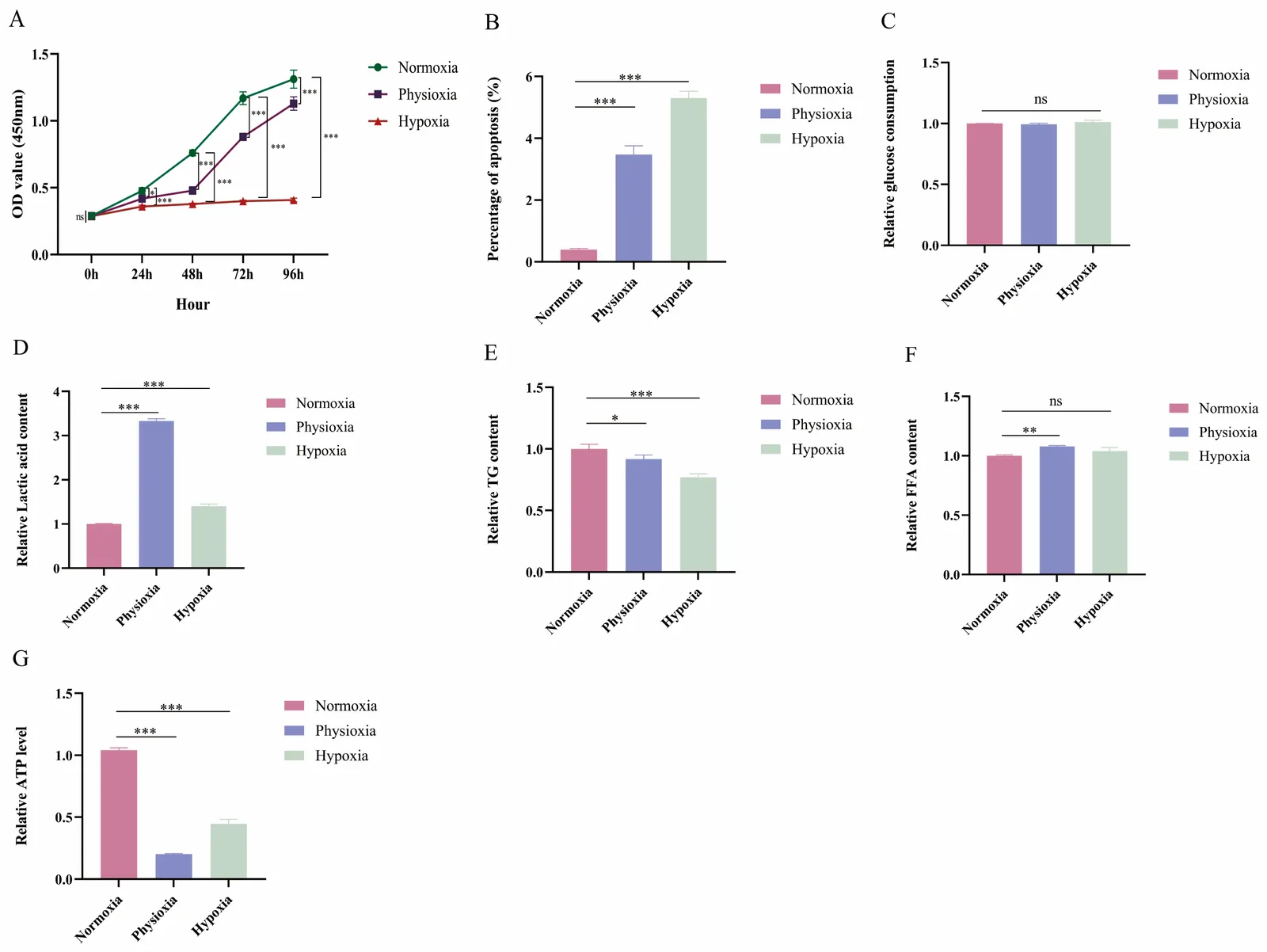
Different oxygen concentrations affect yak subcutaneous preadipocytes. The conditions tested are normoxia, physioxia, and hypoxia, all without exogenous ADM treatment. (**A**) CCK-8 measures changes in cell proliferation between 0 and 96 h. (**B**) Flow cytometry measures apoptosis rates for each group. (**C**) Glucose consumption is measured in each group. (**D**) Relative extracellular lactate levels are measured for each group. (**E**) Relative TG levels are measured in each group. (**F**) Relative FFA levels are measured in each group. (**G**) Relative ATP levels in cells are measured for each group. Data are shown as mean ± SEM. Significant differences are labeled as * *p* < 0.05, ** *p* < 0.01, and *** *p* < 0.001. ns shows no significant difference. Each group includes three replicates (*n* = 3).

In the absence of exogenous ADM interference, we assessed the viability of yak subcutaneous preadipocytes at different oxygen concentrations using the CCK-8 assay. Cell viability varied with oxygen levels: It was significantly lower under physioxia (10% O_2_) than under normoxia (21% O_2_), and the inhibitory effect intensified under hypoxia (1% O_2_) as oxygen concentration decreased (Figure 3A). Flow cytometric apoptosis analysis revealed that both physioxia (10% O_2_) and hypoxia (1% O_2_) significantly induced apoptosis, with the pro-apoptotic effect more pronounced at hypoxia (1% O_2_) (Figure 3B). Assessments of glucose metabolism under different oxygen concentrations revealed no significant differences in relative glucose consumption among groups (Figure 3C). In contrast, analyses of lactate content showed elevated levels in the physioxic (10% O_2_) group compared with the normoxic (21% O_2_) group. Lactate levels in the hypoxic (1% O_2_) group were also higher than in the normoxic (21% O_2_) group but significantly lower than in the physioxic (10% O_2_) group (Figure 3D). For lipid metabolism, TG levels gradually decreased as oxygen concentrations dropped: TG levels were significantly lower in the physioxic (10% O_2_) group than in the normoxic (21% O_2_) group, and this decrease was more pronounced in the hypoxic (1% O_2_) group (Figure 3E). Additionally, analyses of FFA content indicated that extracellular FFA levels were significantly elevated in the physioxic (10% O_2_) group, while FFA levels in the hypoxic (1% O_2_) group showed no significant difference compared to the normoxic (21% O_2_) group (Figure 3F). The ATP assay results showed significantly reduced intracellular ATP levels in both the physioxic (10% O_2_) and hypoxic (1% O_2_) groups compared with the normoxic (21% O_2_) group (Figure 3G). Together, these findings show that physioxia (10% O_2_) is associated with reduced cell proliferation, increased apoptosis, and changes in glycolysis and lipid metabolism, whereas hypoxia (1% O_2_) is associated with further metabolic dysfunction and damage.

### 3.3. Dose-Dependent Regulation of ADM on Proliferation, Migration, and Apoptosis of Yak Subcutaneous Preadipocytes Under Hypoxia

**Figure 4:**
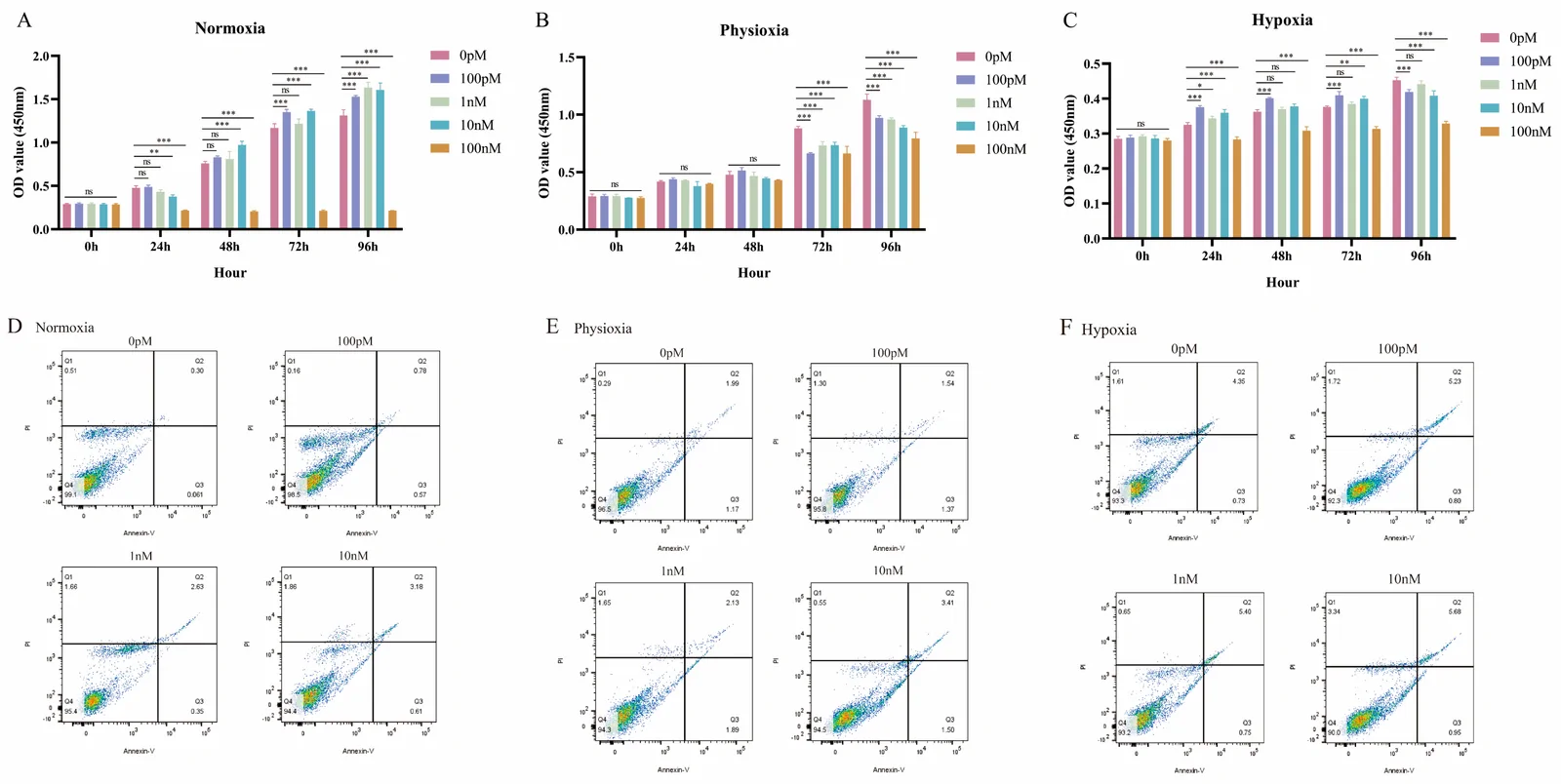
Effects of exogenous ADM on cell proliferation, migration, and apoptosis under normoxia, physioxia, and hypoxia. (**A**–**C**) CCK-8 shows the impact of different ADM concentrations (100 pM–100 nM) on cell proliferation over 0–96 h at various oxygen levels. (**D**–**F**) Flow cytometry analyzes apoptosis after 48 h in the three oxygen conditions with ADM (100 pM–10 nM). Q1 (Annexin V-FITC^−^/PI^+^) indicates necrotic cells; Q2 (Annexin V-FITC^+^/PI^+^) denotes late apoptosis; Q3 (Annexin V-FITC^+^/PI^−^) denotes early apoptosis; Q4 (Annexin V-FITC^−^/PI^−^) marks viable cells. The gradient colors in panels (**D**–**F**) represent the density of cellular events, where warmer colors correspond to higher cell density. * *p* < 0.05, ** *p* < 0.01, and *** *p* < 0.001.

The results of the CCK-8 cell proliferation assay indicate that different oxygen concentrations significantly affect the proliferative capacity of yak subcutaneous preadipocytes. The regulatory effect of exogenous ADM on cell proliferation also exhibits clear concentration dependence and varies with the oxygen environment. Under normoxic (21% O_2_) conditions, cells in the high-concentration ADM (100 nM) treatment group underwent complete cell death. Therefore, the maximum ADM concentration was set to 10 nM for subsequent experiments. Compared to the normoxic condition, the inhibitory effect of the hypoxic (1% O_2_) environment on cell proliferation was significantly stronger than that of physioxia (10% O_2_). Further observations revealed that ADM regulation of cell proliferation under 1% hypoxia exhibited a clear concentration-dependent pattern. Treatment with low-concentration ADM (100 pM) effectively alleviated hypoxia-induced inhibition of cell proliferation within 72 h, restoring proliferative capacity to some extent. In contrast, treatment with high-concentration ADM (10 nM) did not produce a noticeable protective effect, and cell proliferation capacity remained significantly impaired (Figure 4A–C). The results from the cell migration assay further confirmed ADM’s modulatory effect on cell migration under hypoxia (1% O_2_), consistent with the proliferation trends. Under hypoxia (1% O_2_), low-concentration ADM (100 pM) mildly improved cell migration rates and promoted wound healing within 48 h. High-concentration ADM (10 nM) significantly reduced the extent of wound healing (Figure S1A–C). Flow cytometric apoptosis analysis indicated that the apoptosis rate showed a slight upward trend across all ADM-treated groups under normoxic (21% O_2_) conditions, suggesting a mild pro-apoptotic effect of ADM (Figure 4D). Compared with the normoxic control group (21% O_2_), yak subcutaneous preadipocytes showed increased apoptosis under physiological hypoxia (10% O_2_) and hypoxia (1% O_2_). The higher the ADM concentration, the greater the ability to induce apoptosis, with this effect more pronounced under hypoxia (1% O_2_) (Figure 4E,F). These results indicate that a hypoxic (1% O_2_) environment more strongly inhibits cell proliferation and induces apoptosis than a physioxic (10% O_2_) environment. This suggests that yak subcutaneous preadipocytes are highly sensitive to changes in oxygen concentration.

### 3.4. Regulatory Effect of ADM on Lipid Metabolism of Subcutaneous Preadipocytes in Yaks Under Hypoxic Conditions

**Figure 5:**
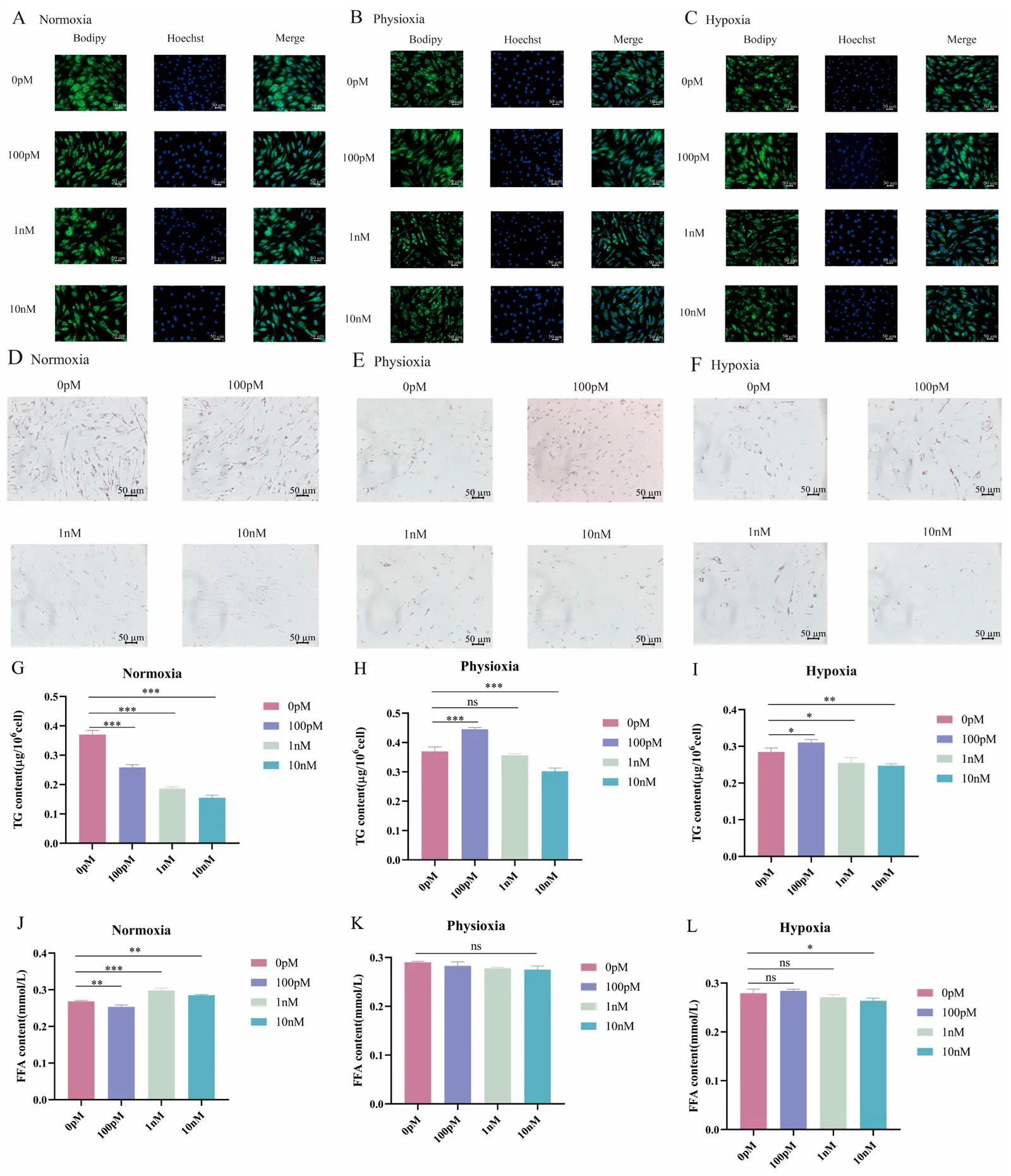
Regulation of lipid metabolic homeostasis in adipocytes by ADM under hypoxic conditions. (**A**–**C**) Bodipy staining visualized lipid droplets (green) and nuclei (blue) in normoxic, physioxia, and hypoxic groups with different ADM concentrations (scale bar = 50 μm). (**D**–**F**) Oil Red O staining showed lipid droplet accumulation in the same groups and conditions (scale bar = 50 μm). (**G**–**I**) Intracellular TG levels. (**J**–**L**) extracellular FFA levels were measured in the respective groups following ADM treatment. Data are the mean ± SEM. Statistical significance: * *p* < 0.05, ** *p* < 0.01, and *** *p* < 0.001; ns, not significant. Each group: three replicates (*n* = 3).

Bodipy results showed that under normoxic conditions, the green fluorescent signal from lipid droplets in the control group was significantly stronger than that in the ADM-treated group. As the ADM concentration increased, lipid droplet accumulation gradually decreased. In the normoxic (21% O_2_) control group, lipid droplet accumulation was higher than in the physioxic (10% O_2_) and hypoxic (1% O_2_) control groups, where accumulation was significantly reduced. ADM exhibited bidirectional, concentration-dependent regulation of lipid droplet formation. Low-concentration ADM (100 pM) alleviated hypoxia-induced inhibition of intracellular lipid droplet accumulation. In contrast, high-concentration ADM (10 nM) still exhibited a strong inhibitory effect (Figure 5A–C). Oil Red O results further indicated that, compared to the normoxic (21% O_2_) control group, lipid droplet staining signals were weaker in both the physioxic (10% O_2_) and hypoxic (1% O_2_) control groups. Moreover, ADM continued to exhibit bidirectional regulation. Under hypoxic conditions, the low-concentration ADM (100 pM) treatment increased the number of red lipid droplet particles, whereas high-concentration ADM (10 nM) treatment resulted in fewer such particles (Figure 5D–F). Quantitative analyses of Oil Red O confirmed that under physioxia (10% O_2_) and hypoxia (1% O_2_), ADM exerted a bidirectional regulatory effect on lipid droplet accumulation. ADM promoted accumulation at low concentrations, but it inhibited it at high concentrations (Figure S2A–C). Thus, the regulatory effect of ADM on lipid metabolism in yak subcutaneous preadipocytes depends on both the oxygen environment and the drug concentration. ADM promotes lipid droplet accumulation only under low-concentration hypoxic conditions. Measurements of lipid-metabolism-related indicators, specifically TG content, revealed that under normoxia (21% O_2_), intracellular TG levels matched the lipid droplet staining results. ADM treatment significantly inhibited lipid accumulation in yak subcutaneous preadipocytes, with greater inhibition at higher concentrations. Under physioxia (10% O_2_), ADM at low concentrations (100 pM) promoted intracellular TG accumulation and slightly alleviated hypoxia’s inhibitory effect, while high concentrations (10 nM) inhibited TG accumulation. The trend was similar under hypoxic (1% O_2_) conditions (Figure 5G–I). Extracellular FFA assays showed that, under normoxia (21% O_2_), FFA levels were significantly reduced in the low-concentration ADM group (100 pM) compared with the control. In the high-concentration ADM (10 nM) group, extracellular FFA levels were significantly increased. Under physioxia (10% O_2_), FFA levels across all ADM-treated groups decreased slightly. Under hypoxic conditions, extracellular FFA in the high-concentration ADM (10 nM) group was significantly lower than in the control group (Figure 5J–L). These results indicate that hypoxia impairs lipid metabolism in yak subcutaneous preadipocytes. The biological effects of ADM change significantly with oxygen concentration. Under normoxic conditions, ADM generally inhibits lipid accumulation. In a hypoxic (1% O_2_) environment, ADM exhibits bidirectional regulatory characteristics: Low-concentration ADM (100 pM) alleviates hypoxia-induced metabolic damage, promotes lipid synthesis, and provides a protective effect. In the high-concentration ADM (10 nM) group, the significant decrease in extracellular FFA may be associated with enhanced metabolic turnover and energy compensation under hypoxic stress.

### 3.5. Effect of ADM on Glycolysis and ATP Production in Subcutaneous Preadipocytes of Yaks Under Hypoxic Conditions

**Figure 6:**
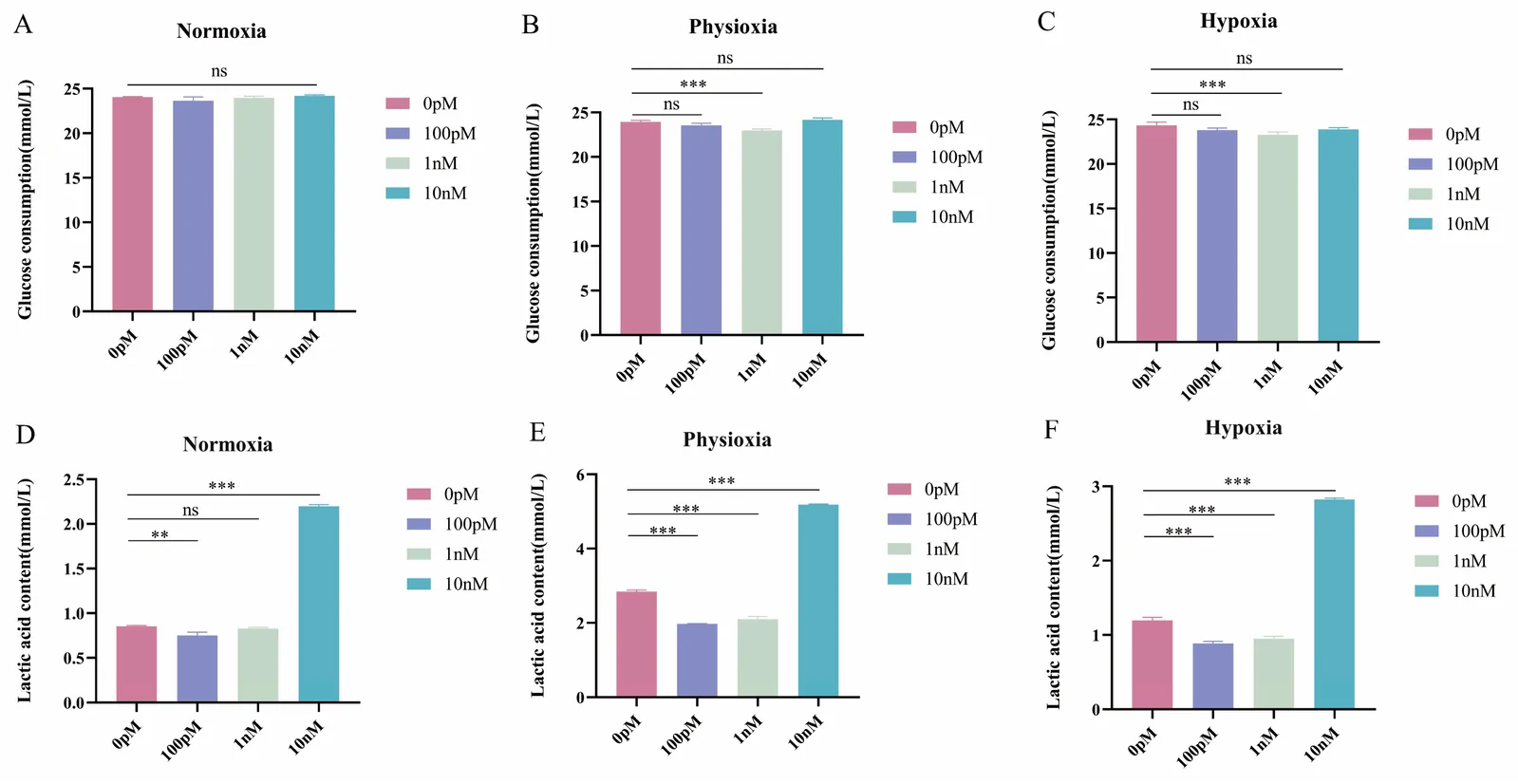

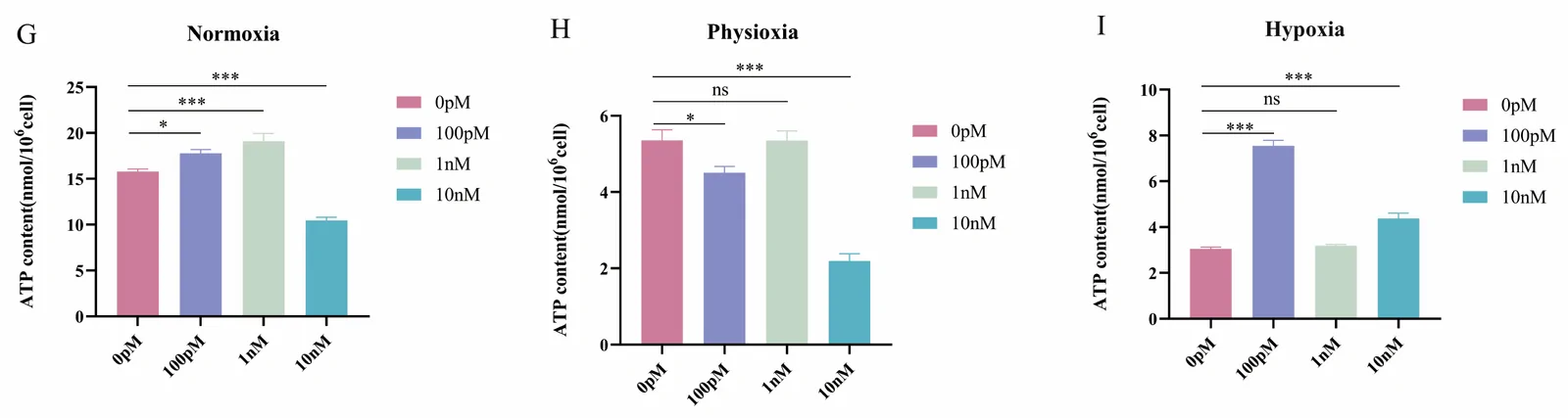
Effects of ADM on glycolysis and ATP production in yak subcutaneous preadipocytes under hypoxia. (**A**–**C**) Measurement of glucose consumption in cells from the normoxic, physioxia, and hypoxic groups under different ADM concentration treatments. (**D**–**F**) Measurement of extracellular lactate levels in cells from the normoxic, physiologically hypoxic, and hypoxic groups under different ADM concentrations. (**G**–**I**) Measurement of ATP production levels in cells from the normoxic, physiologically hypoxic, and hypoxic groups under different ADM concentrations. Data are presented as mean ± SEM. Statistical significance is denoted as follows: * *p* < 0.05, ** *p* < 0.01, and *** *p* < 0.001; ns indicates no significant difference. Each group included three replicates (*n* = 3).

The results of glucose consumption assays showed that, in both the physioxic (10% O_2_) and hypoxic (1% O_2_) groups, only the group treated with medium-concentration ADM (1 nM) exhibited a significant reduction in cellular glucose consumption. No significant differences were observed in the other groups (Figure 6A–C). The results from extracellular lactate level assays revealed that, under physioxia (10% O_2_), ADM exerted bidirectional, concentration-dependent regulation of extracellular lactate levels. Low-concentration ADM (100 pM) inhibited extracellular lactate secretion, while high-concentration ADM (10 nM) promoted its release. Under hypoxia (1% O_2_), the trend was similar to that observed under physioxia (10% O_2_) (Figure 6D–F). ATP content measurements confirmed that ADM exhibits a differential regulatory pattern on ATP production in yak subcutaneous preadipocytes under hypoxic conditions. Under physioxia (10% O_2_), ADM (100 pM and 10 nM) treatment decreased total intracellular ATP levels. Along with changes in lactate secretion, this suggests that overall cellular energy production efficiency is reduced at this time, leading to a decrease in ATP content. In contrast, under hypoxia (1% O_2_), ATP levels in the ADM-treated groups (100 pM and 10 nM) increased, consistent with the trend toward enhanced lactate secretion. This suggests that, under hypoxia (1% O_2_), high-concentration ADM (10 nM) may compensate by maintaining energy supply through glycolysis (Figure 6G–I). In summary, under physioxia (10% O_2_), the low-concentration ADM (100 pM) treatment group exhibited reduced lactate secretion and decreased ATP production, with increased lipid droplet accumulation. This suggests that the cell’s metabolic flux may have partially shifted from glycolytic energy production to lipid synthesis. In contrast, under hypoxia (1% O_2_), the high-concentration ADM (10 nM) treatment group exhibited increased lactate secretion, suggesting enhanced glycolysis. This may represent an important compensatory mechanism for cells responding to extreme energy stress. Together with the results showing decreased extracellular FFA levels and no increase in lipid droplets or TG, these findings suggest that FFA may be taken up by cells and participate in metabolic turnover and energy compensation. Thus, basal energy supply may be maintained through dual reprogramming of glycolysis and fatty acid metabolism.

### 3.6. Analysis of Differential Gene Expression Characteristics in the Transcriptome

**Figure 7:**
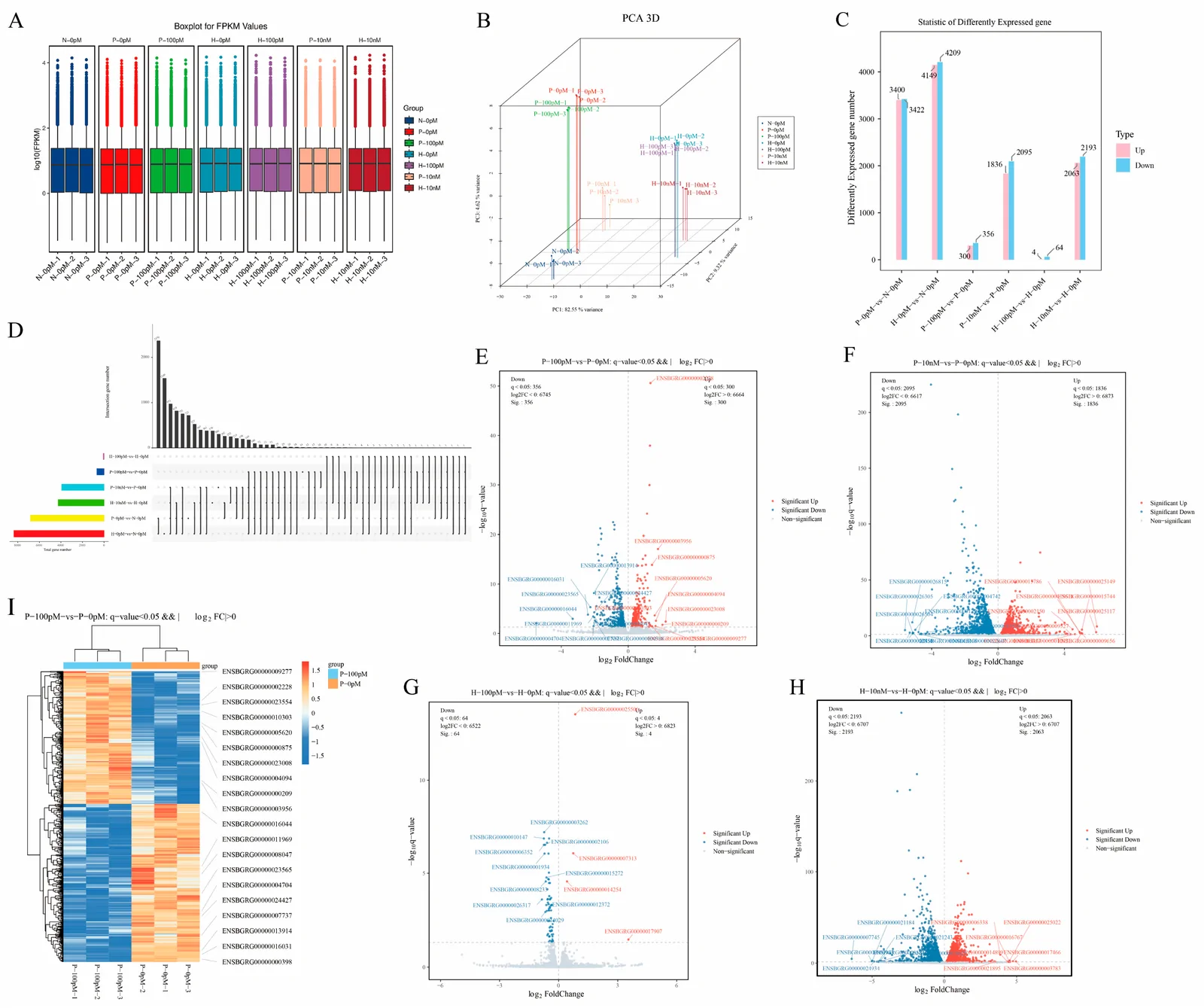

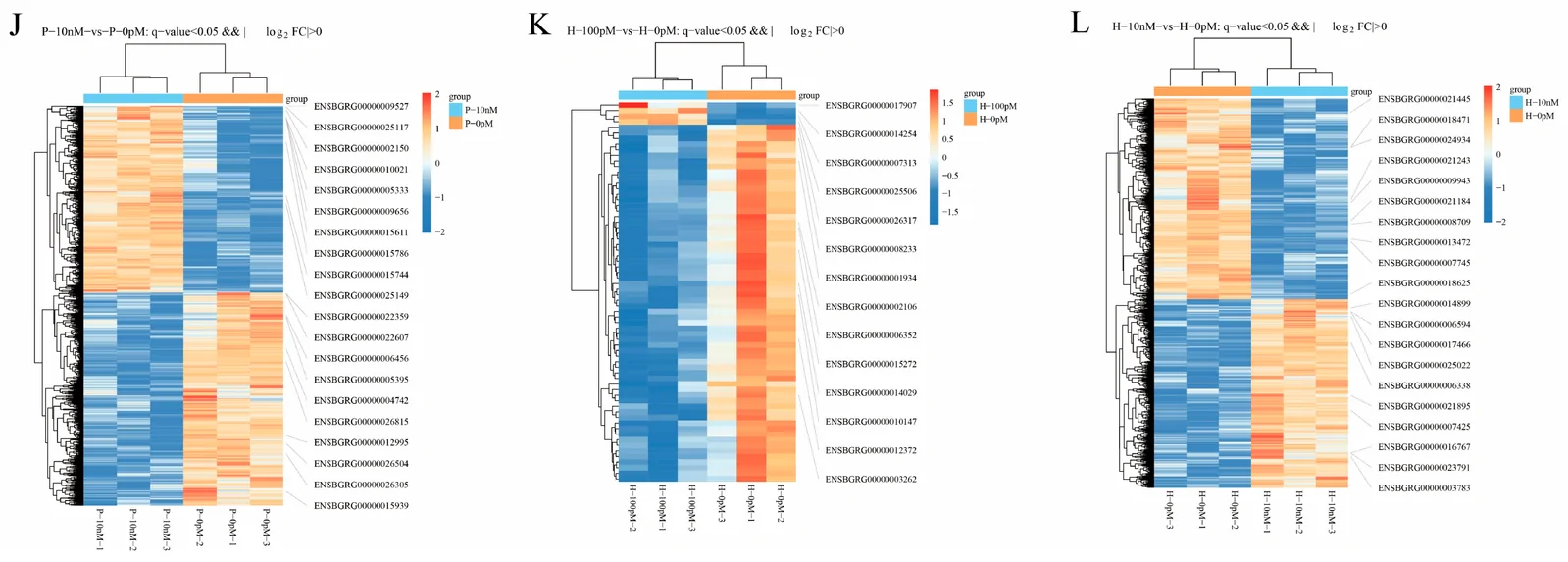
Gene expression profiles and distribution patterns of differentially expressed genes across groups. (**A**) Box plot of gene expression FPKM. The x-axis shows experimental samples in each group, and the y-axis shows gene expression levels (log_10_^FPKM^). The box plots display the distribution and dispersion of gene expression, with five key statistical indicators listed from top to bottom: maximum, upper quartile, median, lower quartile, and minimum. (**B**) PCA. The similarities and differences in gene expression profiles across sample groups are shown. PC1, PC2, and PC3 explain 82.55%, 9.32%, and 4.62% of the variance, respectively. (**C**) Bar chart showing the number of differentially expressed genes. (**D**) UpSet analysis of differentially expressed genes. The horizontal axis shows the number of genes. Each colored bar represents a comparison group, and the bar length indicates the total number of differentially expressed genes in that group. Each column shows a distinct set of gene intersections. A single dot shows that a group has no overlap with other sets, representing genes unique to that group. Horizontal lines connecting black dots indicate that multiple groups share common genes, reflecting shared genes across comparison groups. (**E**–**H**) Volcano plots. These plots display the distribution of DEGs across groups. Gray indicates non-significantly differentially expressed genes; red and blue indicate significantly differentially expressed genes. (**I**–**L**) DEG clustering plots. The horizontal axis shows sample names. Red blocks indicate genes with relatively high expression, while blue blocks indicate genes with relatively low expression. The symbol "&&" represents the logical AND operator, which means both screening thresholds must be satisfied simultaneously.

To elucidate the transcriptional mechanisms by which ADM regulates energy metabolism under hypoxic conditions, this study utilized transcriptomic sequencing to analyze expression patterns in key signaling pathways and differentially expressed genes. Analyses of gene expression distribution (fragments per kilobase of transcript per million mapped reads, FPKM) across all samples revealed that median gene expression values were highly consistent across the seven treatment groups. Distribution patterns in the three biological replicates within each group also overlapped, suggesting high data quality, good reproducibility, and no systematic bias (Figure 7A). Principal component analysis (PCA) further assessed expression similarity among samples. The PCA results showed that biological replicates clustered closely within each group, while samples across groups were clearly separated by oxygen concentrations (normoxia, physioxia, and hypoxia). This suggests that changes in oxygen concentration are likely the primary driver of cellular transcriptomic differences. ADM intervention modulated overall transcriptional profiles (Figure 7B). Statistical analyses of differentially expressed genes showed that a change in oxygen concentration alone, as in the comparison between hypoxia (1% O_2_) (H-0 pM) and normoxia (21% O_2_) (N-0 pM), led to the greatest number of differentially expressed genes: 4149 upregulated and 4209 downregulated. In the comparison between physioxia (10% O_2_) (P-0 pM) and normoxia (21% O_2_) (N-0 pM), 3400 genes were upregulated, and 3422 were downregulated. This indicates a stronger regulatory effect at hypoxia (1% O_2_). Under the same oxygen conditions, ADM treatment exhibited a concentration-dependent effect. The high-concentration ADM (10 nM) group showed far more differentially expressed genes than the 100 pM group, indicating stronger transcriptomic regulation at higher ADM levels (Figure 7C). Overlap analyses of differentially expressed genes with UpSet plots revealed that, under physioxia (10% O_2_) and hypoxia (1% O_2_), genes induced by ADM (10 nM) overlapped substantially with those induced by the hypoxia-only treatment. This suggests that ADM may participate in the transcriptional regulation of cellular hypoxia response pathways. The ADM-treated group also exhibited unique differentially expressed genes that did not overlap with hypoxia-responsive genes, suggesting that ADM may have independent transcriptional regulatory mechanisms under hypoxic conditions (Figure 7D). Volcano plots further analyzed gene expression changes across groups. Both physioxia (10% O_2_) and hypoxia (1% O_2_) stress induced significant changes in gene expression compared to normoxia (21% O_2_). Under hypoxia (1% O_2_), the number of upregulated and downregulated genes was higher, with larger fold changes and greater significance. This indicates that stronger hypoxia evokes a more pronounced transcriptional response (Figure S3A,B). Under the same hypoxic conditions, the low-concentration ADM (100 pM) group exhibited weaker changes in gene expression and fewer differentially expressed genes. The high-concentration ADM (10 nM) group induced widespread upregulation and downregulation of genes under both physioxic (10% O_2_) and hypoxic (1% O_2_) conditions, with more pronounced differences in gene expression (Figure 7E–H). The heatmap of differentially expressed genes showed clear distinctions among the normoxic (21% O_2_), physioxic (10% O_2_), and hypoxic (1% O_2_) groups. After hypoxia treatment, differentially expressed genes formed distinct clusters of upregulated and downregulated modules. Replicates within each group exhibited strong cluster cohesion and consistent expression trends (Figure 7I–L and Figure S3C,D).

### 3.7. GO and KEGG Enrichment Analysis

**Figure 8:**
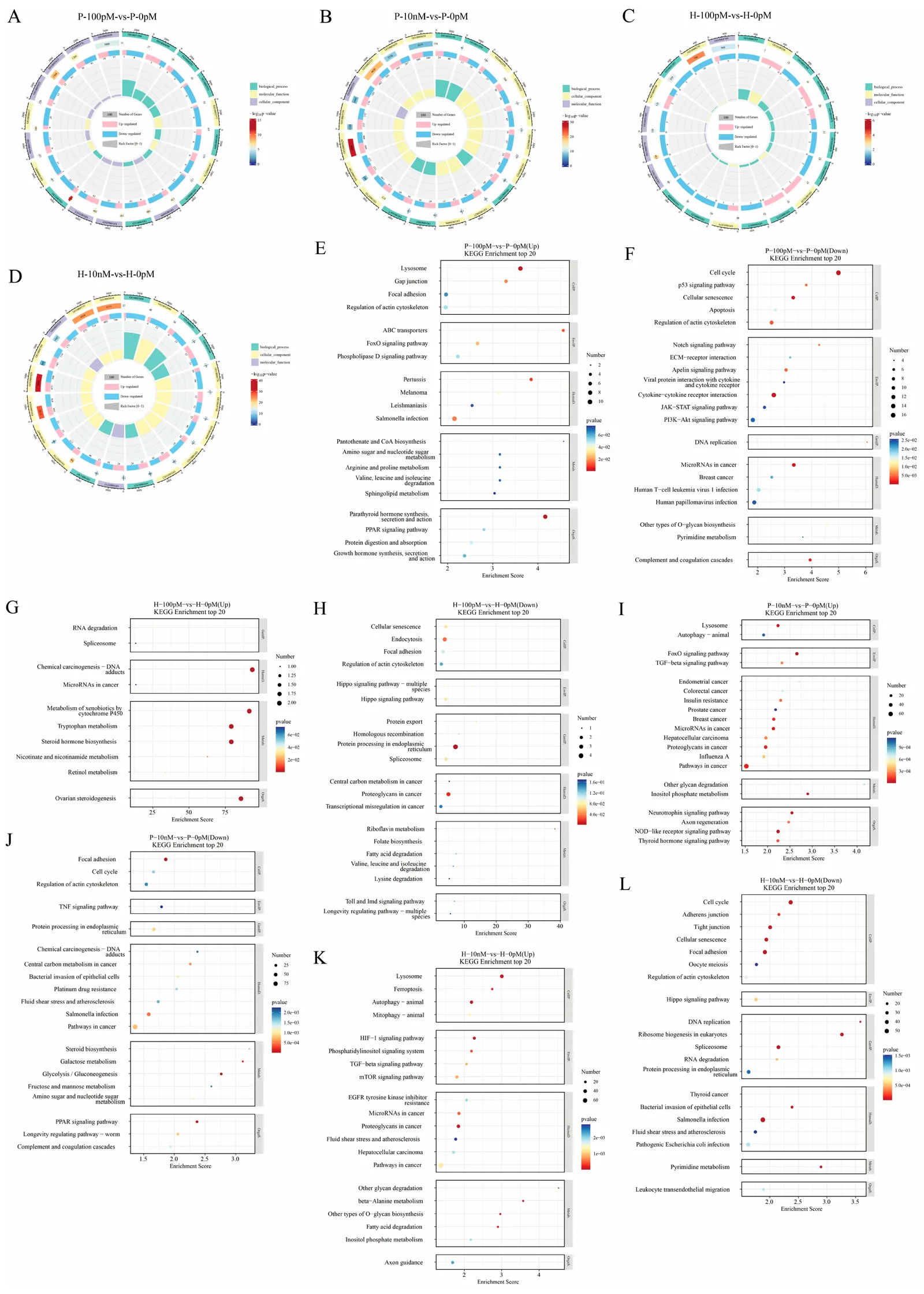
Enrichment analysis. (**A**–**D**) GO enrichment analysis pie charts. The first layer shows functional category annotations. The outer ring scale indicates the number of genes. Each functional category is shown in a different color. The second layer shows the total number of background genes in each category, as well as the enrichment significance. Bar length represents gene count. The color gradient reflects *p*-value levels: Redder means higher enrichment significance, and bluer means lower. The third ring displays the proportion of upregulated and downregulated differentially expressed genes. Light red indicates upregulated genes; light blue indicates downregulated genes. The fourth ring shows the enrichment factor (Rich Factor) for each functional category. (**E**–**L**) KEGG enrichment analysis bubble plots: The horizontal axis represents the enrichment score. The size of the points shows the number of enriched genes in each pathway. The color gradient from blue to red indicates *p*-values from high to low, with enrichment significance increasing from low to high.

The GO functional enrichment clustering results indicate that different oxygen concentrations and ADM treatment exert significantly different effects on the transcriptional regulation of yak subcutaneous preadipocytes. Only the hypoxia (1% O_2_) treatment group induced widespread enrichment of many differentially expressed genes across cellular components, biological processes, and molecular functional pathways. This effect was more pronounced under hypoxic (1% O_2_) conditions (Figure S4A,B). Under the same physioxic (10% O_2_) conditions, ADM treatment exhibited a clear concentration-dependent effect. Low-concentration ADM (100 pM) caused only slight enrichment of a small number of genes. High-concentration ADM (10 nM), however, led to significant enrichment of differentially expressed genes in multiple core functional pathways. This concentration-dependent effect was even more pronounced under hypoxia (1% O_2_) conditions. Here, biological processes and cell-component-related pathways associated with differentially expressed genes showed higher enrichment levels. It is speculated that high-concentration ADM may synergize with the hypoxic environment to significantly alter cellular transcriptional expression profiles and functional states by regulating multiple key functional pathways (Figure 8A–D). To further elucidate the functional regulatory networks of differentially expressed genes in yak subcutaneous preadipocytes under varying oxygen concentrations and ADM intervention, KEGG pathway enrichment analysis was performed on all differentially expressed genes across the comparison groups. The results showed that, in the absence of exogenous ADM intervention, differentially expressed genes in the physioxic (10% O_2_) comparison group (P-0 pM vs. N-0 pM) were enriched in hypoxia-responsive pathways, including the HIF-1 signaling pathway, p53, the MAPK signaling pathway, and other hypoxic stress pathways. In contrast, the upregulated differentially expressed genes in the hypoxia (1% O_2_) comparison group (H-0 pM vs. N-0 pM) were primarily enriched in pathways related to the cell cycle, DNA replication, and damage repair. This finding indicates that cellular transcriptional response patterns differ under varying degrees of hypoxia. Comparing the KEGG enrichment results of downregulated genes between the two groups, the TCA cycle, oxidative phosphorylation, and insulin signaling pathways were significantly enriched in both the physioxic (10% O_2_) and hypoxic (1% O_2_) groups. Compared to the physioxic (10% O_2_) group, the hypoxic (1% O_2_) group exhibited higher enrichment scores for these pathways and a greater number of downregulated genes associated with them. Simultaneously, multiple metabolism-related pathways, including ferroptosis, lysosomes, and endocytosis, were significantly downregulated exclusively in the hypoxic (1% O_2_) group. These results suggest that hypoxia (1% O_2_) exerts a stronger inhibitory effect on aerobic metabolism and insulin signaling. As a result, cellular energy metabolism may shift from aerobic respiration to anaerobic metabolism (Figure S4C–F). In the ADM intervention group, the low-concentration ADM (100 pM) treatment group showed reduced enrichment of energy-metabolism-related pathways, such as glycolipid and amino acid metabolism, under both physioxic (10% O_2_) and hypoxic (1% O_2_) conditions (Figure 8E–H). The high-concentration ADM (10 nM) treatment group exhibited significant regulatory effects on pathways under both physioxia (10% O_2_) and hypoxia (1% O_2_). Under physioxia (10% O_2_), differentially upregulated genes were significantly enriched in the FoxO signaling pathway and insulin-resistance-related pathways. Differentially downregulated genes were enriched in glucose metabolism pathways, such as glycolysis and gluconeogenesis, and the PPAR signaling pathway (Figure 8I–L).

### 3.8. Protein–Protein Interaction Network (PPI)

**Figure 9:**
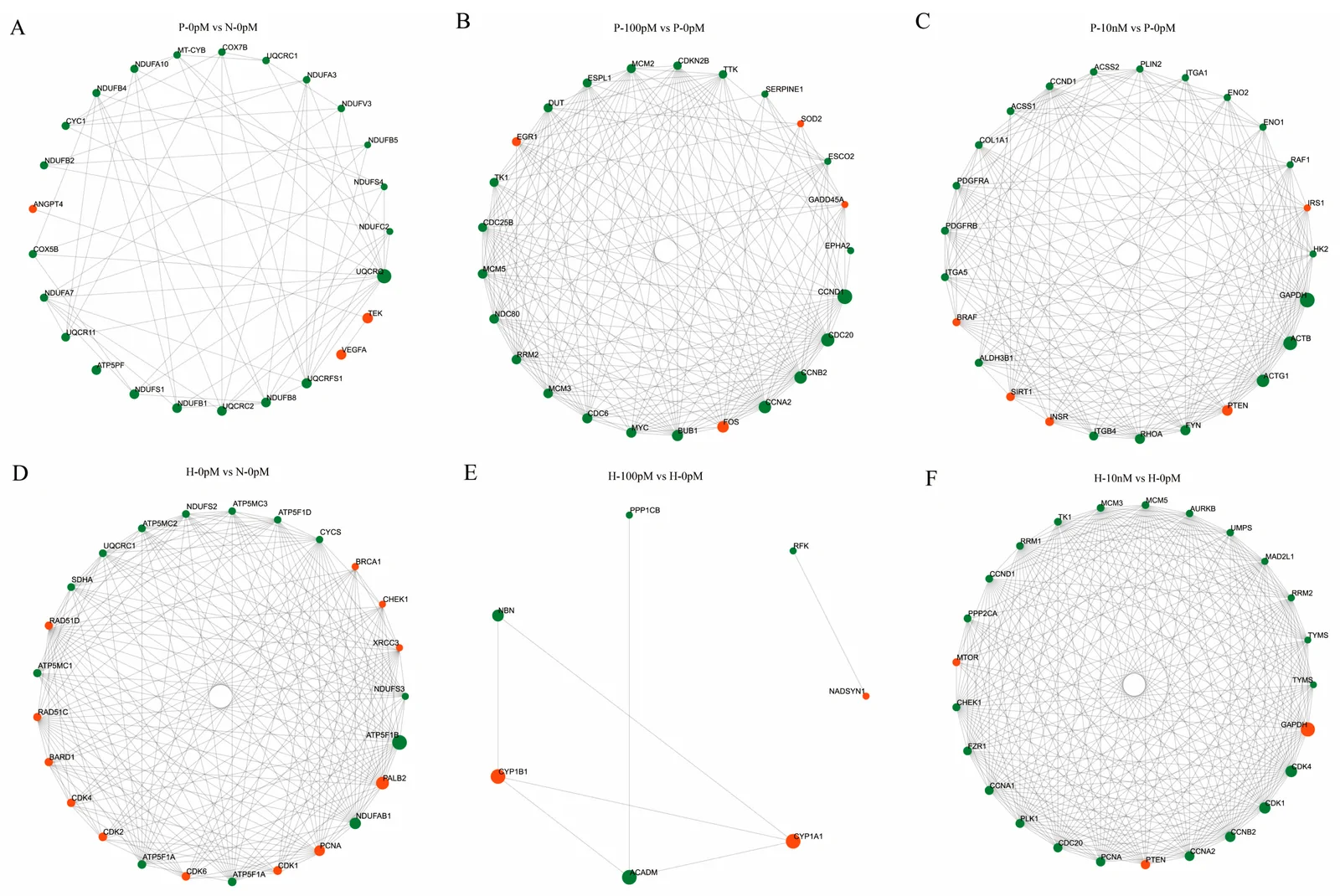
PPI network. (**A**–**F**) Core hub genes related to energy metabolism, hypoxic response, cellular stress, and cell proliferation were identified from each comparison group based on the top 25 nodes ranked by connectivity, and the network was plotted using gene names. In the figure, circles represent differentially expressed genes; red indicates upregulated genes, and green indicates downregulated genes. The size of the circle represents the degree of connectivity; a larger circle indicates a higher degree of connectivity for that gene.

In each comparison group, key differentially expressed genes related to energy metabolism, hypoxic response, cellular stress, and cell proliferation were identified. These genes were used to construct a PPI network to elucidate the interaction patterns and regulatory relationships among core genes under different treatment conditions. The results showed that under varying oxygen concentrations and ADM treatment, the regulatory patterns of PPI functional modules changed with the gradient of culture conditions. They exhibited a stepwise differential response. Under physioxia (10% O_2_), compared with the normoxic (21% O_2_) group, the network core was dominated by genes involved in oxidative phosphorylation (*NDUF, UQCR,* and *COX* families). This was accompanied by an enrichment of hypoxic stress response genes such as *ANGPT4* and *VEGFA*. These findings indicate that physioxia (10% O_2_) primarily triggers cellular hypoxia sensing and adaptation of basal metabolism (Figure 9A). The network structure of the low-concentration ADM (100 pM) comparison group was relatively simple. It was dominated by cell-cycle- and proliferation-related genes, with only a few stress-response genes, such as FOS and EGR1, enriched. This indicates that low-concentration ADM induces only mild stress and basic homeostasis regulation (Figure 9B). The network structure of the high-concentration ADM (10 nM) comparison group was complex. It was simultaneously enriched for key genes involved in glycolysis and energy metabolism (*ENO1*, *ENO2*, and *HK2*), regulatory genes for lipid metabolism (*PLIN2* and *ACSS* family), and genes involved in the insulin signaling pathway (*IRS1* and *INSR*). This indicates that high-concentration ADM significantly regulates energy metabolism and stress response pathways (Figure 9C). Under hypoxic (1% O_2_) conditions, compared to the normoxic (21% O_2_) group, the network centered on mitochondrial respiratory chain complex genes (*ATP5* family and *NDUF* family). It was also enriched with DNA damage repair and cell-cycle-related genes (*BRCA1, CHEK1*, and *RAD51* family). This indicates that hypoxia (1% O_2_) primarily initiates mitochondrial metabolic inhibition and cellular damage defense programs (Figure 9D). The network in the low-concentration ADM (100 pM) comparison group had fewer nodes. It was dominated by genes involved in metabolic detoxification and oxidative stress, such as *CYP1A1* and *CYP1B1*, with no significant enrichment of energy-metabolism-related pathways. This indicates that low-concentration ADM, under hypoxia (1% O_2_), primarily participates in cellular stress defense and maintenance of homeostasis, without initiating significant remodeling of energy metabolism (Figure 9E). The network structure of the high-concentration ADM (10 nM) comparison group was the most complex. It was centered on cell-cycle regulatory genes (*MCM* family, *CCN* family, and *CDK* family) and incorporated key energy-metabolism nodes such as *GAPDH*, *mTOR,* and *PTEN*. This indicates that high-concentration ADM can act synergistically with hypoxia to jointly mediate cell cycle arrest and energy metabolism reprogramming (Figure 9F).

Taken together with the KEGG enrichment results, the data indicate that the dominant pathways across treatment groups exhibit distinct, condition-dependent differences in their responses. Under physioxia (10% O_2_), energy metabolism predominated. Low-concentration ADM under hypoxic (1% O_2_) conditions favored stress defense responses. High-concentration ADM treatment significantly triggered energy metabolism reprogramming. Consequently, the absence of enrichment for energy-metabolism-related genes in some groups led to differences in pathway selection when cells responded to hypoxic stress of varying intensities.

### 3.9. Key Gene Verification

**Figure 10:**
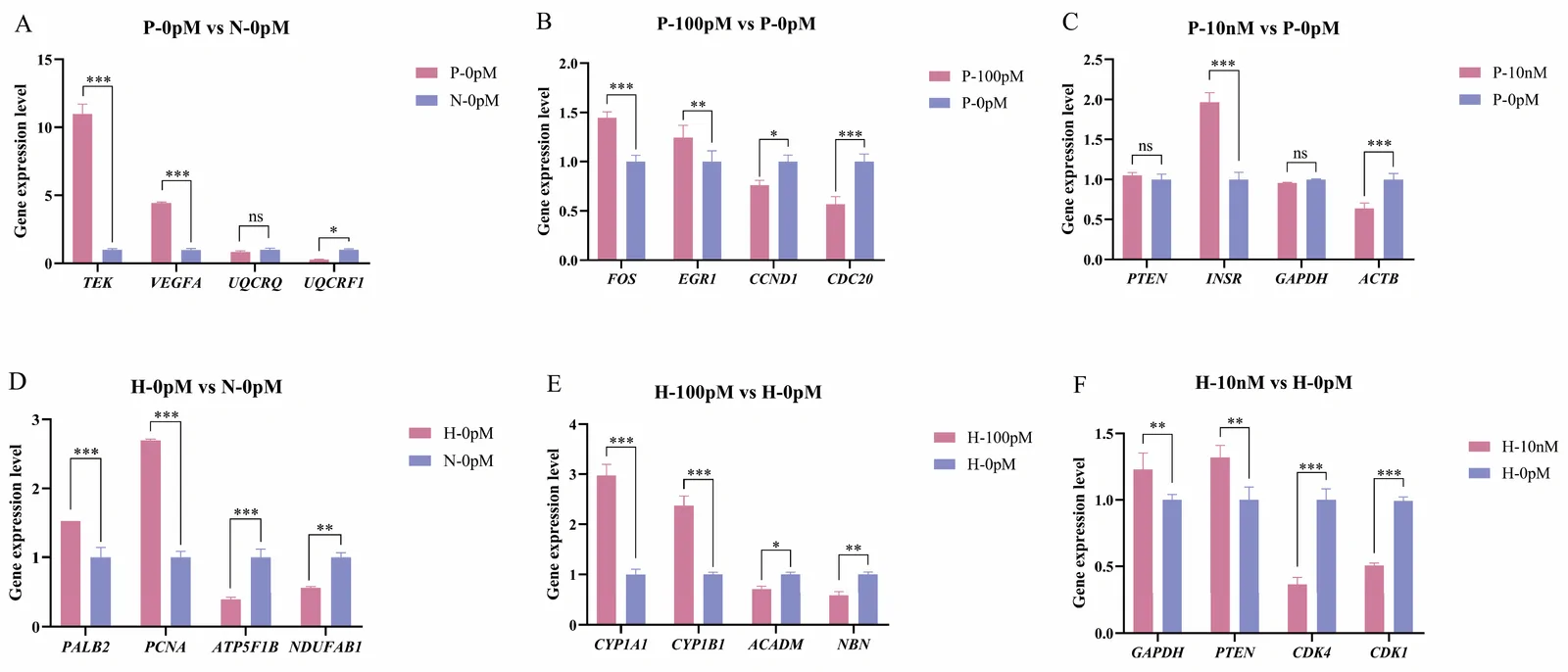
mRNA expression levels of relevant genes across comparison groups. (**A**–**F**) mRNA expression levels of relevant genes in cells from the normoxic group, physioxia group, and hypoxic group after 48 h of treatment with ADM at different concentrations. Data are presented as mean ± SEM. Statistical significance compared to the normoxic group is indicated as follows: * *p* < 0.05, ** *p* < 0.01, and *** *p* < 0.001; ns indicates no significant difference; three replicates were performed for each group (*n* = 3).

Based on the protein interaction network results, node connectivity was the primary screening criterion. Hub genes from each comparison group were selected for qPCR validation to clarify the expression trends in core genes. Compared with the normoxic (21% O_2_) group (N-0 pM), the expression levels of the hypoxia-responsive genes *TEK* and *VEGFA* were significantly elevated in the physioxic (10% O_2_) group (P-0 pM). The expression of the mitochondrial-respiratory-chain-related gene *UQCRF1* was significantly decreased. This indicates that physioxia (10% O_2_) significantly activates the hypoxic stress pathway and suppresses the expression of genes related to mitochondrial aerobic respiration (Figure 10A). In the low-concentration ADM (P-100 pM) treatment group, the expression of FOS and *EGR1*—key early genes regulating cellular stress and proliferation—was significantly upregulated. The expression of cell-cycle-related genes *CCND1* and *CDC20* was suppressed (Figure 10B). In the high-concentration ADM (P-10 nM) treatment group, the insulin signaling pathway gene *INSR* was significantly upregulated, while the cytoskeletal-related gene *ACTB* was significantly downregulated (Figure 10C). Under hypoxic (1% O_2_) conditions, compared with the normoxic (21% O_2_) control group (N-0 pM), the expression levels of DNA-damage-repair-related genes *PALB2* and *PCNA* were significantly elevated in the hypoxia (1% O_2_) treatment group (H-0 pM). The expression levels of mitochondrial-respiratory-chain-related genes *ATP5F1B* and *NDUFAB1* were significantly reduced. This indicates that hypoxia can activate cellular damage-defense responses and suppress genes involved in mitochondrial aerobic respiration (Figure 10D). In the low-concentration ADM (H-100 pM) treatment group, the metabolic detoxification and oxidative stress genes *CYP1A1* and *CYP1B1* were significantly upregulated. The fatty-acid-metabolism-related gene ACADM and the DNA-repair-related gene NBN were significantly downregulated (Figure 10E). In the high-concentration ADM (H-10 nM) treatment group, the expression levels of *GAPDH*, a key gene in glycolysis, and *PTEN*, a key gene in the mTOR pathway, were significantly increased. The expression levels of cell-cycle-related genes *CDK4* and *CDK1* were significantly decreased (Figure 10F). The expression trends of these key genes were generally consistent with the transcriptomic sequencing results. This further validated the reliability of the sequencing data. Additionally, the functional differences in the differentially expressed genes across the comparison groups further confirmed that preadipocytes from yak hide follow a stepwise adaptation pattern. They prioritize stress responses, while metabolic remodeling lags in response to hypoxia (1% O_2_) and ADM intervention.

## 4. Discussion

To the best of our knowledge, this study is the first to systematically explore the concentration-dependent bidirectional regulatory effects of adrenomedullin (ADM) on proliferation, apoptosis, glycolipid metabolism, and migration of yak subcutaneous preadipocytes under different oxygen gradients. Our core findings are summarized as follows: (1) Hypoxia significantly inhibited the proliferation of yak subcutaneous preadipocytes and induced apoptosis, and the inhibitory effect was enhanced with the decrease in oxygen concentration. (2) Under hypoxic conditions, ADM exerted bidirectional regulation on preadipocyte fate and metabolism in a concentration-dependent manner: low-dose ADM (100 pM) partially alleviated hypoxia-induced proliferation inhibition and metabolic damage, while high-dose ADM (10 nM) further aggravated proliferation suppression and promoted apoptosis.

High-altitude hypoxia is a core environmental stress that restricts the physiological metabolism of plateau animals. As a key energy storage and endocrine organ with high metabolic plasticity, adipose tissue plays a critical role in animals’ adaptation to high-altitude hypoxia and cold stress [17]. Oxygen homeostasis directly determines the proliferation, differentiation, and survival of preadipocytes. Accumulated evidence from previous studies has confirmed that hypoxia can comprehensively reshape the metabolic phenotype and cellular fate of adipocytes by inhibiting mitochondrial aerobic respiration, blocking ATP synthesis, and inducing oxidative stress and apoptosis, which represents a universal adaptive response of mammalian adipocytes to low-oxygen environments [18,19,20]. As an endemic species long adapted to high-altitude hypoxia and cold stress, yaks have evolved unique hypoxic metabolic regulatory mechanisms in their subcutaneous adipocytes, which maintain energy homeostasis under hypoxia by dynamically regulating glycolysis, lipid synthesis, and fatty acid degradation pathways. In this study, we used yak subcutaneous preadipocytes as an in vitro model to investigate the regulatory role of ADM in hypoxic adaptation. We systematically confirmed that ADM exerts concentration-dependent bidirectional regulation on cell fate and glycolipid metabolism of preadipocytes under hypoxic stress. Our findings revealed the regulatory pattern of yak high-altitude hypoxic adaptation from multiple dimensions, including cell proliferation, apoptosis, glycolysis, ATP production, and lipid accumulation, and identified ADM as an important mediator of hypoxic metabolic adaptation in yak subcutaneous preadipocytes.

The dynamic balance between cell proliferation and apoptosis is fundamental to maintaining the homeostasis of preadipocyte numbers and ensuring the normal development and metabolic function of adipose tissue. Environmental stressors and interventions with bioactive factors can directly disrupt this balance, inducing cell cycle arrest, inhibition of proliferation, and even programmed cell death [21]. Previous studies have confirmed that a hypoxic microenvironment can significantly inhibit DNA replication and cell division in various cell types, induce cell-cycle arrest, and reduce cell proliferation [22,23]. Adrenomedullin (ADM), as a key environmental response factor, exerts broad regulatory effects on cell proliferation and apoptosis. It promotes stem cell proliferation and inhibits apoptosis by activating pathways such as PI3K/Akt, thereby maintaining cellular homeostasis [24,25]. The results of this study indicate that the inhibitory effect of hypoxia on the proliferative activity of yak subcutaneous preadipocytes is oxygen-concentration-gradient-dependent; both physioxia (10% O_2_) and hypoxia (1% O_2_) significantly inhibit cell proliferation and promote apoptosis, with the stress effect of hypoxia (1% O_2_) being more pronounced. The core mechanism may be that hypoxia (1% O_2_) significantly inhibits mitochondrial aerobic respiration, resulting in insufficient ATP to support DNA replication and cell cycle progression, thereby inducing cell cycle arrest and cellular damage. More importantly, this study confirms that the regulation of adipocyte proliferation and apoptosis by ADM under hypoxic (1% O_2_) conditions exhibits a significant concentration-dependent bidirectional pattern: Low-concentration ADM (100 pM) can effectively alleviate hypoxia-induced proliferation inhibition, exert a positive protective effect, and maintain basic cellular physiological activity under hypoxia. In contrast, high-concentration ADM (10 nM) showed a significant synergistic inhibitory effect together with hypoxic stress, which markedly downregulated the expression of cell-cycle-promoting positive regulatory genes, including *CDK1* and *CDK4*. The decreased transcription of these core cyclin-dependent kinase genes indicates suppressed cell cycle progression and aggravated cell cycle arrest, ultimately facilitating the activation of apoptotic programs. From an energy-metabolism perspective, the proliferation suppression triggered by high-concentration ADM serves as a vital energy-conserving adaptive strategy for cells under hypoxia. Repression of energy-consuming events, including cell division and anabolism, reduces overall oxygen and ATP expenditure; allows cells to prioritize fundamental homeostatic maintenance; and ultimately improves the adaptive tolerance to persistent low-oxygen surroundings.

Energy metabolism reprogramming is the core adaptive mechanism by which adipocytes respond to hypoxic stress and is also the key mechanism by which ADM regulates the hypoxic phenotype of cells [26]. Under normoxic conditions, adipocytes primarily rely on the mitochondrial tricarboxylic acid cycle and oxidative phosphorylation to achieve efficient aerobic energy production; under hypoxic stress, aerobic metabolic pathways are impaired, forcing cells to initiate metabolic compensation by upregulating anaerobic glycolysis to rapidly generate ATP, resulting in the typical hypoxic metabolic feature of “suppressed aerobic metabolism and enhanced anaerobic metabolic compensation” [27,28]. Previous studies have shown that ADM family factors are extensively involved in regulating cellular energy homeostasis. ADM can inhibit mitochondrial respiratory function by regulating the activity of key enzymes in glycolysis, and this regulatory effect exhibits significant concentration-dependent differences [29,30]. This study confirms that, under different oxygen partial pressure conditions, ADM can regulate cellular energy status by reshaping the flow of carbohydrate and lipid metabolism, exhibiting distinct metabolic regulation patterns. Under physioxia (10% O_2_), cells exhibit the classic Warburg effect [31], characterized by massive lactate accumulation, a significant drop in ATP levels, and a pronounced compensatory glycolytic response. In contrast, under hypoxia (1% O_2_), the glycolytic pathway is restricted, and cellular metabolic focus shifts from glycolysis to fatty acid degradation to compensate for energy supply, forming a unique hypoxia-adaptive metabolic pattern. Building on this, ADM further amplifies the bidirectional metabolic regulation observed under hypoxic conditions: Low-concentration ADM (100 pM) effectively reduces glycolytic intensity, decreases abnormal lactate accumulation, moderately restores cellular ATP supply, and inhibits wasteful anaerobic metabolic losses. Simultaneously, it redirects more metabolic substrates toward lipid synthesis and triglyceride accumulation, promoting lipid droplet formation and driving cells into a protective metabolic steady state characterized by low energy expenditure and high energy storage; in contrast, the effects of high-concentration ADM (10 nM) exhibit distinct patterns at the transcriptional and functional levels: KEGG enrichment analysis of the transcriptome revealed overall downregulation of genes associated with glycolysis and gluconeogenesis pathways, suggesting that high-concentration ADM (10 nM) suppresses the expression of glycolysis-related genes at the transcriptional level; however, cellular functional assays detected a significant increase in lactate levels. It is hypothesized that high-concentration ADM treatment enhances cellular glucose uptake (activation of the insulin resistance pathway). Concurrently, hypoxia inhibits mitochondrial oxidative phosphorylation, preventing the influx of glucose into the TCA cycle. Consequently, glucose is forced to be converted into lactate via the glycolytic pathway, leading to elevated lactate levels. Additionally, key rate-limiting enzymes in glycolysis are regulated by post-translational modifications; even under transcriptional downregulation, they can maintain metabolic flux by increasing their enzymatic activity. Furthermore, downregulation of the PPAR pathway reduces the diversion of carbon sources toward lipid synthesis, ultimately resulting in transcriptional suppression of glycolytic genes and, at the functional level, massive lactate production. High concentrations of ADM (10 nM) synergize with hypoxia to further inhibit mitochondrial aerobic respiration, functionally driving enhanced anaerobic metabolism and exacerbating energy metabolism disorders while simultaneously driving the massive uptake and utilization of free fatty acids to compensate for the energy deficit under hypoxia through dual carbohydrate–lipid compensatory pathways.

This study also found that yak subcutaneous preadipocytes exhibit a stepwise adaptive strategy in response to ADM under hypoxic conditions. At physioxia (10% O_2_), cells prioritize metabolic adaptation to maintain energy balance; at hypoxia (1% O_2_), cells shift toward stress defense to ensure survival. Under the synergistic effect of hypoxia (1% O_2_) and high-concentration ADM, cells undergo systemic metabolic reprogramming. This pattern clearly demonstrates that yak subcutaneous preadipocytes dynamically switch response modes in response to oxygen concentration and ADM dose, which may also explain why energy metabolism pathways were not enriched in some comparison groups in the transcriptomic sequencing analysis.

Lipid metabolic remodeling is a key metabolic strategy for yak subcutaneous preadipocytes to adapt to the high-altitude hypoxic environment, and the formation of lipid droplets and the level of triglyceride accumulation directly determine the cells’ energy storage capacity and environmental adaptability [32]. This study confirms that ADM regulation of lipid metabolism in hypoxic adipocytes also exhibits a strict concentration-dependent bidirectional pattern: Low concentrations of ADM (100 pM) significantly promote lipid droplet formation and triglyceride accumulation, enhancing cellular lipid energy storage capacity, while high-concentration ADM (10 nM) significantly inhibits lipid synthesis, blocks lipid droplet accumulation, and promotes fatty acid degradation for energy production; this regulatory pattern closely aligns with the bidirectional regulatory characteristics of ADM in mouse preadipocytes [33]. At the transcriptional level, transcriptome enrichment analysis indicated that ADM might remodel the glucose–lipid metabolic network through multiple core signaling pathways with divergent trends under different concentrations. Bioinformatic prediction suggested that low-concentration ADM tended to enrich the PPAR signaling pathway, which was annotated to potentially modulate adipogenic differentiation and lipid storage via the PPARγ-C/EBPα-FABP4 transcriptional axis to maintain hypoxic metabolic homeostasis; nevertheless, the PPAR pathway showed downregulated enrichment in several pairwise comparisons, reflecting inconsistent pathway responses across treatment gradients. On the contrary, high-concentration ADM treatment exhibited significant enrichment of the HIF-1α-mediated hypoxic response pathway, which was predicted to crosstalk with the mTOR energy-sensing cascade to jointly drive enhanced glycolysis, fatty acid catabolism, and cell cycle arrest, ultimately shaping the adaptive transcriptional phenotype of cells under sustained hypoxia. It should be noted that these pathway regulatory models are only inferred from RNA-seq enrichment results and require further experimental verification for confirmation [34,35].

Furthermore, this study found that high concentrations of ADM downregulate *ACTB* gene expression; *ACTB* encodes the core actin protein of the cytoskeleton and directly regulates cytoskeletal assembly and structural stability. Previous studies have confirmed that cytoskeletal remodeling participates in the regulation of energy metabolism by modulating mitochondrial spatial distribution, structural integrity, and respiratory chain assembly efficiency [36], suggesting that ADM may indirectly influence mitochondrial function by regulating cytoskeletal homeostasis and thereby contributing to the reprogramming of energy metabolism under hypoxia. This mechanism may provide a new direction for future research.

Combining transcriptomic sequencing results with cellular phenotypic assays reveals that hypoxia globally inhibits oxidative phosphorylation and the TCA cycle in adipocytes, thereby reducing aerobic metabolic efficiency. Concurrently, it suppresses activity in the insulin signaling pathway, reduces cellular sensitivity to glucose metabolism, and induces metabolic stress. ADM serves an important regulatory role in cellular energy metabolism under hypoxic conditions, exerting concentration-dependent divergent metabolic and cellular effects: Low-concentration ADM triggers adaptive metabolic programs that prioritize energy storage and metabolic homeostasis; high-concentration ADM further inhibits aerobic metabolism, activates compensatory fatty acid degradation and glycolysis pathways, and induces cell cycle arrest, enabling cells to adapt to hypoxia via a low-energy-consumption metabolic pattern with enhanced compensatory metabolism [37]. This study is the first to incorporate ADM into the energy metabolism regulatory network of yak subcutaneous preadipocytes under hypoxic conditions, clarifying its core function in concentration-dependent bidirectional regulation of glucose and lipid metabolism and the remodeling of energy homeostasis and thereby refining the theoretical framework for energy metabolism regulation in subcutaneous adipose tissue of high-altitude livestock.

This study systematically elucidated the concentration-dependent mechanisms and core phenotypic characteristics of ADM in regulating energy metabolic remodeling in hypoxic yak subcutaneous preadipocytes; however, certain research limitations remain. First, while the in vitro cellular model used in this study to simulate oxygen partial pressure gradients effectively isolates the regulatory effects of ADM from oxygen environmental variables, it cannot replicate the complex microenvironment and multifactorial interactions within yak adipose tissue, resulting in some deviation between the findings and the actual physiological metabolic state of the animals. Second, while this study focused on elucidating the overall patterns of ADM-mediated reprogramming of glucose and lipid metabolism and associated cellular phenotypic changes, it did not delve into microscopic energy metabolism mechanisms such as mitochondrial respiratory function and mitochondrial dynamic homeostasis; the upstream and downstream molecular regulatory networks remain to be further elucidated. Furthermore, this study used only male yaks as experimental subjects. It did not systematically account for the regulatory effects of confounding factors such as sex, age, and seasonal environmental fluctuations on lipid metabolic phenotypes, which, to some extent, limits the generalizability and ecological relevance of our study’s conclusions. To address these limitations, future research could be deepened from multiple dimensions: specifically, by validating the function of the HIF-1α/mTOR/PPAR signaling axis mediated by ADM and its key receptors and elucidating the regulatory mechanisms governing the remodeling of adipocyte energy metabolism; conducting in vivo ADM intervention trials in yaks to evaluate systematically, at the individual level, the regulatory effects of ADM on fat deposition, energy homeostasis, and high-altitude hypoxia tolerance; and extablishing a dynamic research system encompassing different sexes, ages, and seasons to elucidate the regulatory mechanisms of subcutaneous adipocyte energy metabolism under the interaction of environmental and physiological conditions. By integrating research at the molecular, cellular, and individual levels, we can further improve the regulatory theory of how high-altitude mammals adapt to hypoxia, providing scientific evidence and theoretical support for breeding hardy livestock and healthy farming in high-altitude areas.

## 5. Conclusions

Using yak subcutaneous preadipocytes as a model, this study elucidates how oxygen concentration and ADM jointly drive transcriptional and metabolic responses in these cells, revealing distinct stage-dependent characteristics. Specifically, hypoxia markedly inhibits cell proliferation and facilitates apoptosis, and measurements of glucose consumption and lactate production confirmed elevated glycolysis levels under hypoxic conditions. Under this hypoxic (1% O_2_) condition, ADM exerts a concentration-dependent, bidirectional regulatory effect. At low concentrations (100 pM), ADM, specifically under 1% O_2_ hypoxia, effectively alleviates hypoxia-induced cell proliferation inhibition; elevates intracellular ATP content; and promotes lipid droplet formation and triglyceride accumulation. These changes only represent improvements in partial phenotypic indicators and confer limited metabolic adaptation upon cells exposed to hypoxic stress. By contrast, high-concentration ADM (10 nM) acts synergistically with hypoxia (1% O_2_) to further inhibit aerobic metabolism; activate HIF-1, mTOR, FoxO, and fatty acid degradation pathways; induce cell cycle arrest; and remodel glucose and lipid metabolism. These results demonstrate that yak subcutaneous preadipocytes exhibit distinct phasic characteristics in response to varying oxygen partial pressures and ADM concentrations, with significant metabolic remodeling occurring under hypoxic and high-ADM conditions. Overall, this study improved the theoretical framework of hypoxia adaptation in yak subcutaneous preadipocytes, providing new theoretical foundations and experimental support for research on the low adaptability mechanisms of plateau livestock.

## Data Availability

The transcriptome assembly and raw sequencing data have been deposited in the NCBI Sequence Read Archive (SRA) under the BioProject accession number PRJNA479144.

## Supplementary Materials

The following supporting information can be downloaded at https://www.mdpi.com/article/10.3390/ani16162531/s1. Table S1. Primer sequences provide detailed information for qPCR. Figure S1. Cell migration. (A–C) Cells from the normoxia group, physiological hypoxia group, and hypoxia group were treated with different concentrations of ADM (100 pM–10 nM), and cell migration ability was measured at 0 h, 24 h, 48 h, and 72 h (scale = 200 μm). Figure S2. Oil Red O Quantification. (A–C) OD values for each cell group were measured at 510 nm. Data are shown as mean ± standard error of the mean (SEM). Compared with the normoxia group, statistical significance is indicated as follows: * *p* < 0.05, ** *p* < 0.01, *** *p* < 0.0001, and ns means no significant difference. Each group had three replicates (*n* = 3). Figure S3. Volcano Plot and Cluster Heatmap. (A,B) show volcano plots. Display the distribution of DEGs for the treated and control groups, with gray representing non-significant genes and red and blue representing significantly different genes. (C,D) are DEG clustering plots. The x-axis shows sample names, red blocks indicate genes with relatively high expression, and blue blocks indicate relatively low gene expression. Figure S4. GO and KEGG enrichment analysis. (A,B) GO enrichment analysis circle plot. From the outside in, the first layer shows function-al classification annotation, with the outer scale indicating the number of genes, and different colors representing different func-tional categories. The second layer shows the total number of background genes in each category and the significance of enrich-ment; the length of the bars represents the number of genes, and the color gradient reflects the *p*-value, with redder colors indi-cating higher enrichment significance and bluer colors indicating lower significance. The third circle shows the proportion of up-regulated and downregulated differential genes, with light red and light blue representing upregulated and downregulated gene proportions, respectively. The fourth circle shows the enrichment factor (Rich Factor) for each functional category. (C–F) KEGG en-richment analysis bubble plot. The horizontal axis represents the enrichment score, the size of the dots indicates the number of genes enriched in the pathway, and the color from blue to red represents the *p*-value from high to low (enrichment significance from low to high).

## Abbreviations

The following abbreviations are used in this manuscript:

ADM	Adrenomedullin
HIF	Hypoxia-inducible factor
PBS	Phosphate-buffered saline
P/S	Penicillin–streptomycin
FBS	Fetal bovine serum
DMSO	Dimethyl sulfoxide
CCK-8	Cell Counting Kit-8
GO	Gene Ontology
KEGG	Kyoto Encyclopedia of Genes and Genomes
FPKM	Fragments per kilobase of transcript per million mapped reads
PCA	Principal component analysis
PPI	Protein–protein interaction
TG	Triglyceride
FFA	Free fatty acid
SEM	Standard error of the mean
qPCR	Quantitative polymerase chain reaction
DEGs	Differentially expressed genes
ANOVA	One-way analysis of variance
