# Supplementary material for: Regulatory Role of Adrenomedullin in Hypoxic Adaptation of Yak Subcutaneous Preadipocytes
Source: Animals (Basel). 2026 Aug 13;16(16):2531. doi: 10.3390/ani16162531 (PMC13508979; doi:10.3390/ani16162531)
Supplement: Supplementary file 1 [file animals-16-02531-s001.zip › animals-4453385-supplementary.pdf]

Supplementary Material

Regulatory Role of Adrenomedullin in Hypoxic Adaptation of Yak Subcutaneous Preadipocytes

Su Shan, Hui Jiang, Jincheng Zhong, Yuqing Zhang, Heru Zhang, Zhixin Chai *

Supplementary Tables

Table S1. Primer sequences provide detailed information for qPCR

Genes	Gene ID	Forward primer (5'-3')	Reverse primer (5'-3')
UXT	XM_005899362.3	GCGCTACGAGGCTTTCATCT	GCTTCCTGGAGTCGCTCAAT
CD34	XM_070384702.1	TCTGCCTGAAGCTAAATGA-GACC	TCGGCCTGTTTCTTCTGACAC
ITGB1	XM_070380978.1	GTGAATGTGAGTGCCAGGGT	TGTCTTCGCTGTTCACCTCAT
FABP4	XM_005897260.3	ACAGGAAAGTCAAGAGCATCGT	TCATGACACATTCCAGCACCA
PPARγ	XM_070359416.1	GCGCAGTTGTTCAGGTTTGA	GTCAACCATGGTAATTTT-GTTCA
ATP5F1B	XM_005903447.2	CTACTGTGTTGTCCCGTGCT	ATGATGCGAGACGTGGAGTC
NDUFAB1	XM_005911972.3	GCCAGTATAGCGATGCACCA	CCAAACTGTCTAAGCCCAGGT
PALB2	XM_005910900.3	CTTACACCCAGGCACCCTAC	ACGTGGCACTCATATCCACA
PCNA	XM_005906528.3	GAGGGCTTCGACACTTACCG	TGCCAACGTGTCTGCGTTAT
GAPDH	XM_070371805.1	TGGTGAAGGTCGGAGTGAAC	ATTGATGGCGACGATGTCCA
CDK4	XM_005901664.2	GCTTGCCAGTGGAGACCATA	GCTTGACTGTCCCACCACTT
CDK1	XM_070364572.1	TTTGGCCTTGCCAGAGCTT	TGGAGTTGAGTAGCGAGCTG
PTEN	XM_070363759.1	CATTGCCTGTGTGTGGTGAC	GGTTTCCTCTGGTCCTGGTATG
ACADM	XM_070367848.1	GTGGGACTAGCACAGAGAGC	ATTGCCATTTCAGCCAGCAA
NBN	XM_070382921.1	TTTCAGCCGCTTACCTCTCC	GAACAAGAC-GTTTCCATTCTTGC
CYP1B1	XM_014477564.2	CCAGCCAGGATACTCTCTCCA	GATCCAGTTCTGCCTGTACCC
CYP1A1	XM_014481458.2	ACTTCATCCCTGTCCTCCGT	GTGATGTCCCGGATGTGACC
UQCRQ	XM_005890430.3	CTACTTCAGCAAGGGCATCC	TTCTCAAACTCCTGCGTTCC
TEK	XM_005904802.2	TCATCGCTTGGACCCTTAGC	GCCCTGAACCTTGTAACGGA

VEGFA	XM_070361294.1	GGTACATTGGAGCCTTGCCT	GCGCTGGTAGACATCCATGA
UQCRFS1	XM_005910801.3	CCTTTGGTCGCTTCCGTGA	CTAACACTTCAGGGCGACGAT
ACTB	XM_070362169.1	GCGGCATTCACGAAACTACC	TGTTGGCGTAGAGGTCCTTG
INSR	XM_070373711.1	CAAGGAACCCAACGGTCTGA	GCATAATGTCGGCGGGAGA
CCND1	XM_070360197.1	GCACGACTTCATCGAGCACTTC	AGGGCGGGTTGGAAATGAACT
CDC20	XM_070368319.1	GCACAAGTGACCGACACATTC	GACCAGAGGATGGAGCACAC
FOS	XM_005900989.3	CAAGCGGAGACAGACCAACT	GAGCCGCTAGGATGAACTCG
EGR1	XM_005891717.3	CTTCGCCTGTGACATCTGTG	CGCAAGTGGATCTTGGTATGC

Supplementary Figures

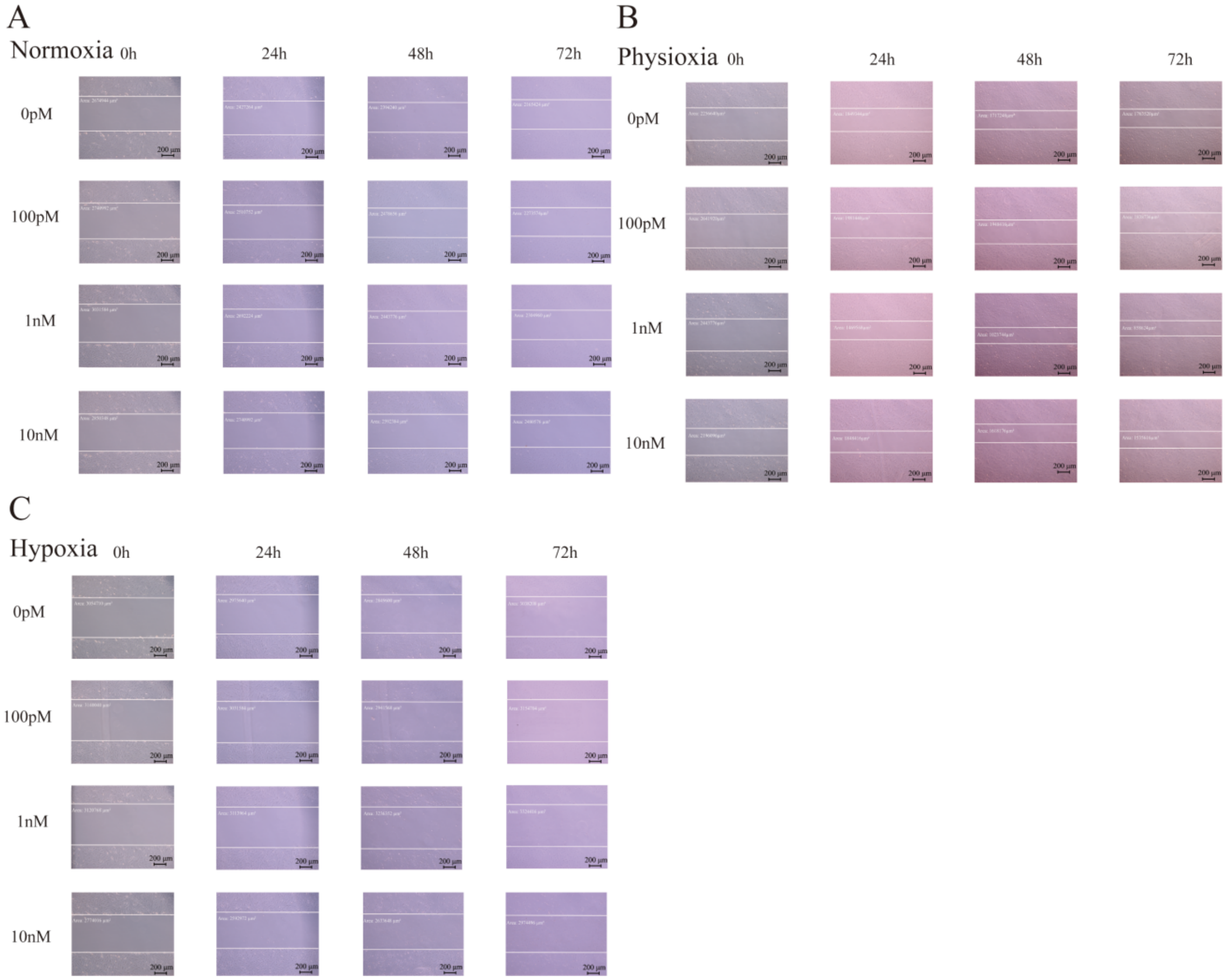

Figure S1. Cell migration. A-C Cells from the normoxia group, physiological hypoxia group, and hypoxia group were treated with different concentrations of ADM (100 pM-10 nM), and cell migration ability was measured at 0 h, 24 h, 48 h, and 72 h (scale = 200 μm);

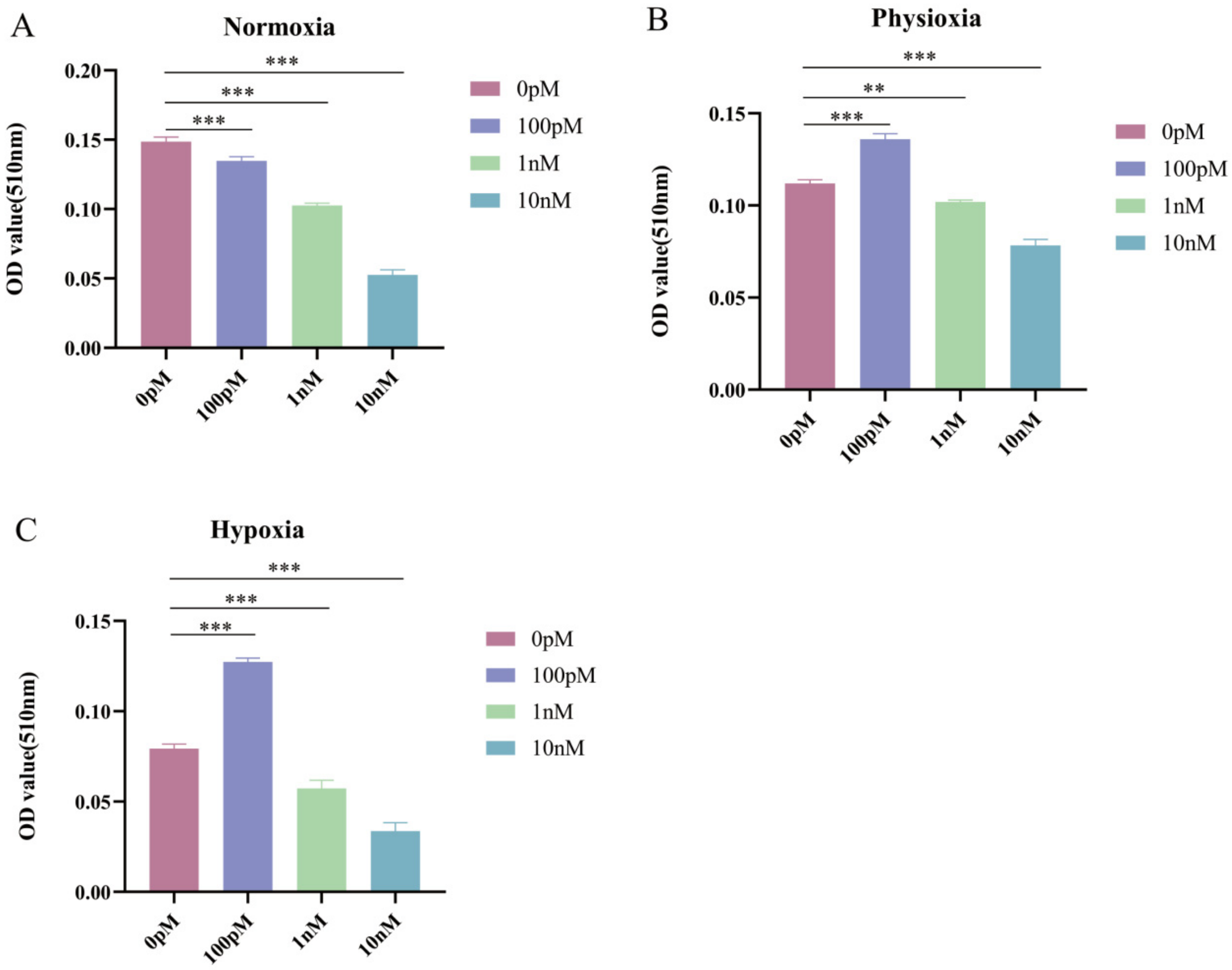

Figure S2. Oil Red O Quantification. A-C OD values for each cell group were measured at 510 nm. Data are shown as mean ± standard error of the mean (SEM). Compared with the normoxia group, statistical significance is indicated as follows: *p < 0.05, **p < 0.01, ***p < 0.0001, and ns means no significant difference. Each group had three replicates (n=3).

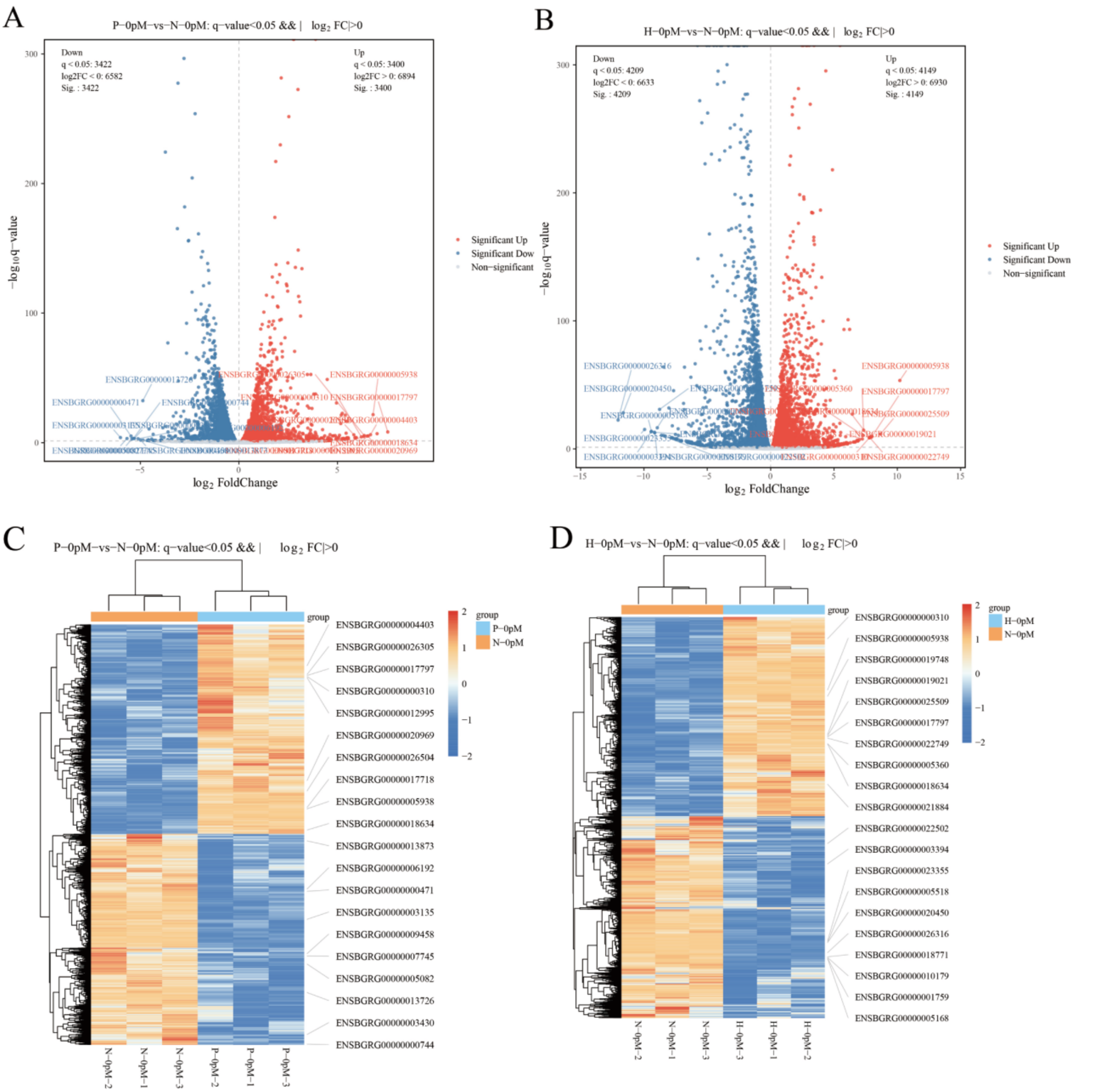

Figure S3. Volcano Plot and Cluster Heatmap. A and B show volcano plots. Display the distribution of DEGs for the treated and control groups, with gray representing non-significant genes and red and blue representing significantly different genes. C and D are DEG clustering plots. The x-axis shows sample names, red blocks indicate genes with relatively high expression, and blue blocks indicate relatively low gene expression.

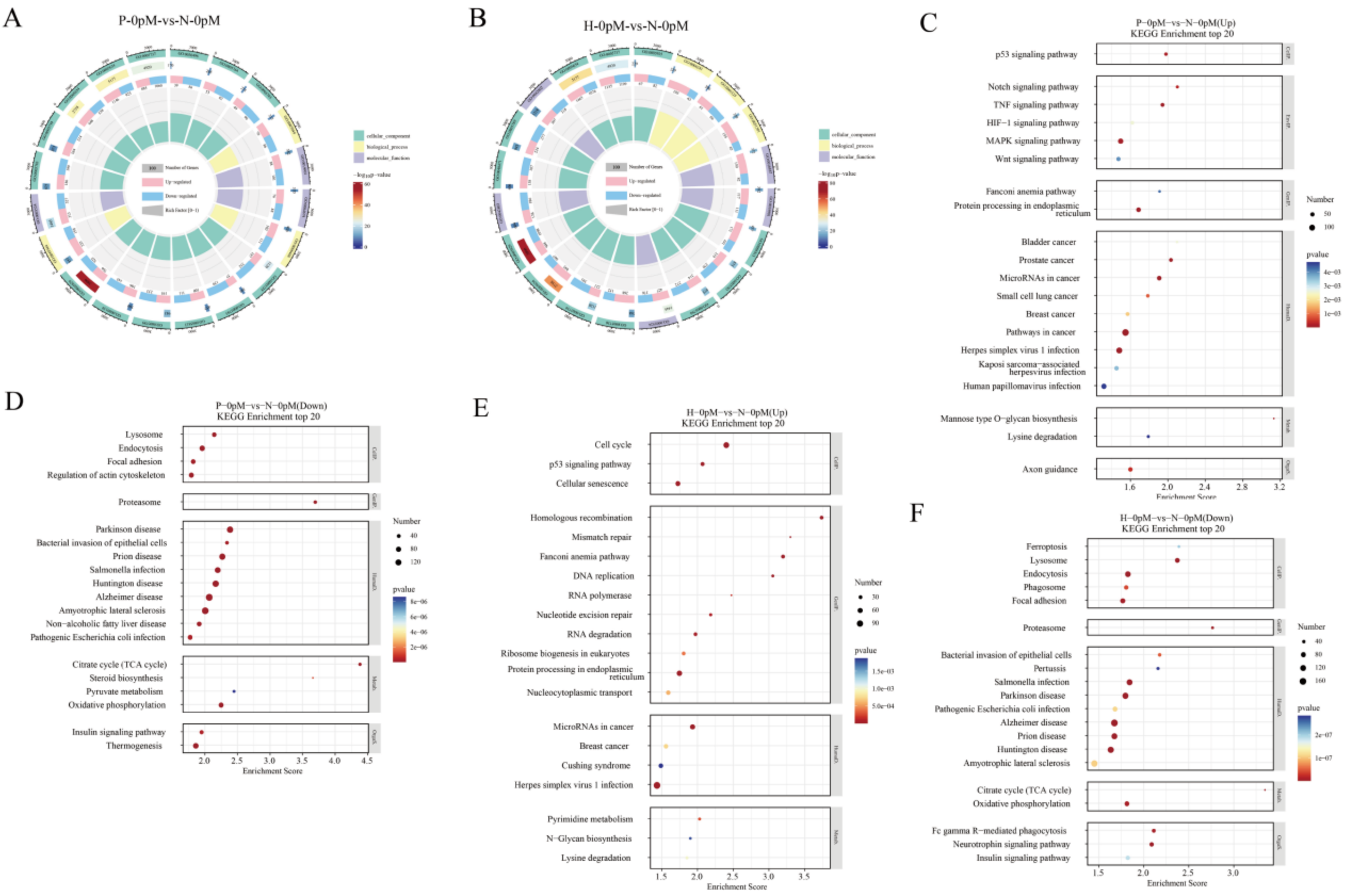

Figure S4. GO and KEGG enrichment analysis. A and B GO enrichment analysis circle plot. From the outside in, the first layer shows functional classification annotation, with the outer scale indicating the number of genes, and different colors representing different functional categories. The second layer shows the total number of background genes in each category and the significance of enrichment; the length of the bars represents the number of genes, and the color gradient reflects the p-value, with redder colors indicating higher enrichment significance and bluer colors indicating lower significance. The third circle shows the proportion of upregulated and downregulated differential genes, with light red and light blue representing upregulated and downregulated gene proportions, respectively. The fourth circle shows the enrichment factor (Rich Factor) for each functional category. C-F KEGG enrichment analysis bubble plot. The horizontal axis represents the enrichment score, the size of the dots indicates the number of genes enriched in the pathway, and the color from blue to red represents the p-value from high to low (enrichment significance from low to high).
